# Biosynthesis of β-(1→5)-Galactofuranosyl Chains of Fungal-Type and *O*-Mannose-Type Galactomannans within the Invasive Pathogen Aspergillus fumigatus

**DOI:** 10.1128/mSphere.00770-19

**Published:** 2020-01-15

**Authors:** Yuria Chihara, Yutaka Tanaka, Minoru Izumi, Daisuke Hagiwara, Akira Watanabe, Kaoru Takegawa, Katsuhiko Kamei, Nobuyuki Shibata, Kazuyoshi Ohta, Takuji Oka

**Affiliations:** a Department of Applied Microbial Technology, Faculty of Biotechnology and Life Science, Sojo University, Kumamoto, Japan; b Department of Infection and Host Defense, Tohoku Medical and Pharmaceutical University, Sendai, Japan; c Graduate School of Environmental and Life Science, Okayama University, Okayama, Japan; d Medical Mycology Research Center, Chiba University, Chiba, Japan; e Department of Bioscience and Biotechnology, Faculty of Agriculture, Kyushu University, Fukuoka, Japan; Carnegie Mellon University

**Keywords:** *Aspergillus fumigatus*, cell wall, glycosyltransferase, galactomannan, galactofuranose, glycosylation, galactofuranosyltransferase

## Abstract

The pathogenic fungus Aspergillus fumigatus contains galactomannans localized on the surface layer of its cell walls, which are involved in various biological processes. Galactomannans comprise α-(1→2)-/α-(1→6)-mannan and β-(1→5)-/β-(1→6)-galactofuranosyl chains. We previously revealed that GfsA is a β-galactofuranoside β-(1→5)-galactofuranosyltransferase involved in the biosynthesis of β-(1→5)-galactofuranosyl chains. In this study, we clarified the biosynthesis of β-(1→5)-galactofuranosyl chains in A. fumigatus. Two paralogs exist within A. fumigatus: GfsB and GfsC. We show that GfsB and GfsC, in addition to GfsA, are β-galactofuranoside β-(1→5)-galactofuranosyltransferases by biochemical and genetic analyses. GfsA, GfsB, and GfsC can synthesize β-(1→5)-galactofuranosyl oligomers at up to lengths of 7, 3, and 5 galactofuranoses within an established *in vitro* highly efficient assay of galactofuranosyltransferase activity. Structural analyses of galactomannans extracted from Δ*gfsB*, Δ*gfsC*, Δ*gfsAC*, and Δ*gfsABC* strains revealed that GfsA and GfsC synthesized all β-(1→5)-galactofuranosyl residues of fungal-type and *O*-mannose-type galactomannans and that GfsB exhibited limited function in A. fumigatus. The loss of β-(1→5)-galactofuranosyl residues decreased the hyphal growth rate and conidium formation ability and increased the abnormal hyphal branching structure and cell surface hydrophobicity, but this loss is dispensable for sensitivity to antifungal agents and virulence toward immunocompromised mice.

**IMPORTANCE** β-(1→5)-Galactofuranosyl residues are widely distributed in the subphylum Pezizomycotina of the phylum Ascomycota. Pezizomycotina includes many plant and animal pathogens. Although the structure of β-(1→5)-galactofuranosyl residues of galactomannans in filamentous fungi was discovered long ago, it remains unclear which enzyme is responsible for biosynthesis of this glycan. Fungal cell wall formation processes are complicated, and information concerning glycosyltransferases is essential for understanding them. In this study, we showed that GfsA and GfsC are responsible for the biosynthesis of all β-(1→5)-galactofuranosyl residues of fungal-type and *O*-mannose-type galactomannans. The data presented here indicate that β-(1→5)-galactofuranosyl residues are involved in cell growth, conidiation, polarity, and cell surface hydrophobicity. Our new understanding of β-(1→5)-galactofuranosyl residue biosynthesis provides important novel insights into the formation of the complex cell wall structure and the virulence of the members of the subphylum Pezizomycotina.

## Abstract (precis)

β-(1→5)-Galactofuranosyl residues are widely distributed in the subphylum Pezizomycotina of the phylum Ascomycota. Pezizomycotina includes many plant and animal pathogens. Although the structure of β-(1→5)-galactofuranosyl residues of galactomannans in filamentous fungi was discovered long ago, it remains unclear which enzyme is responsible for biosynthesis of this glycan. Fungal cell wall formation processes are complicated, and information concerning glycosyltransferases is essential for understanding them. In this study, we showed that GfsA and GfsC are responsible for the biosynthesis of all β-(1→5)-galactofuranosyl residues of fungal-type and *O*-mannose-type galactomannans. The data presented here indicate that β-(1→5)-galactofuranosyl residues are involved in cell growth, conidiation, polarity, and cell surface hydrophobicity. Our new understanding of β-(1→5)-galactofuranosyl residue biosynthesis provides important novel insights into the formation of the complex cell wall structure and the virulence of the members of the subphylum Pezizomycotina.

## INTRODUCTION

The cell wall of the pathogenic fungus Aspergillus fumigatus comprises several kinds of polysaccharides, including chitin, β-(1→3)-glucan, β-(1→3)-/β-(1→4)-glucan, α-(1→3)-glucan, galactosaminogalactan, and galactomannans (GMs) (1–3). These polysaccharides are complexly intertwined to form the three-dimensional structure of cell walls (1, 2). GMs are polysaccharides comprising d-mannose (Man) and d-galactofuranose (Gal*_f_*), localized on the surface layer of cell walls (2), and delineated into fungal-type galactomannan (FTGM) and *O*-mannose (*O*-Man)-type galactomannan (OMGM) (4). FTGM includes core mannan, a structure wherein α-(1→2)-mannotetraose is linked with an α-(1→6)-linkage from position 9 to position 10 and with β-(1→5)-/β-(1→6)-galactofuran side chains (5, 6). FTGM is bound to a glycosylphosphatidylinositol anchor as a carrier molecule and transported from the Golgi apparatus to the cell surface via the secretory pathway (7), and the transported FTGM is incorporated into the β-(1→3)-glucan–chitin core of the cell wall by *DFG* family proteins (8). The OMGM structure comprises β-(1→5)-/β-(1→6)-galactofuranosyl chains bonded to an *O*-Man-type glycan with a structure wherein Man is bonded to serine/threonine of a protein as a basic skeleton (6, 9).

Information on GM biosynthesis has been more thoroughly investigated recently (3, 10, 11). CmsA/Ktr4 has been reported to be an α-(1→2)-mannosyltransferase involved in the biosynthesis of the α-(1→2)-mannan backbone of FTGM (12, 13). In the gene-disrupted *cmsA*/*ktr4* strain and/or the homologous *cmsB*/*ktr7* strain, pronounced hyphal elongation suppression and conidium formation failure were previously observed (12, 13). Moreover, the Δ*cmsA*/*ktr4* mutant was significantly less virulent than the parental strain (13). These data indicate that FTGM is crucial for normal cell growth and virulence (12, 13). GfsA was first identified as a galactofuranosyltransferase involved in the biosynthesis of OMGM galactofuranosyl residues (14). GfsA is a β-galactofuranoside β-(1→5)-galactofuranosyltransferase also involved in the biosynthesis of FTGM galactofuran side chains (4). However, in the Δ*gfsA* strain of A. fumigatus, the β-(1→5)-galactofuranosyl residue was not completely lost (4). The biosynthesis characteristics of the remaining β-(1→5)-galactofuranosyl residues remain unclear. Therefore, we focused on clarifying which residual β-(1→5)-galactofuranosyl residues are biosynthesized. There are two paralogs in A. fumigatus, namely, GfsB and GfsC. We evaluated whether GfsB and GfsC are responsible for biosynthesis of the remaining β-(1→5)-galactofuranosyl residues. We obtained recombinant proteins of GfsA, GfsB, and GfsC to elucidate galactofuranoside chain biosynthesis activity *in vitro* using an established highly efficient assay of galactofuranosyltransferase activity. Furthermore, to investigate the function of *gfs* family proteins *in vivo*, we analyzed the structure of GM extracted from single, double, and triple gene disruptants of *gfsA*, *gfsB*, and *gfsC*. In this study, we aimed to clarify the biosynthesis and function of β-(1→5)-galactofuranosyl residues in A. fumigatus.

## RESULTS

### Features of GfsB and GfsC in A. fumigatus.

The Δ*gfsA* disruptant exhibited reduction of the content of β-(1→5)-galactofuranosyl residues within FTGM and OMGM (4); however, these residues remained within the galactomannan fractions. To determine the enzyme synthesizing the remaining β-(1→5)-galactofuranosyl residues, we focused on *gfsA* paralogs, termed *gfsB* (A. fumigatus 4g13710 [Afu4g13710] in A. fumigatus; Af293/AFUB_070620 in A. fumigatus A1163) and *gfsC* (Afu4g10170/AFUB_067290). Comparison of cDNA and genome sequences revealed that, similarly to *gfsA*, no introns were present in *gfsB* and *gfsC* (4, 14). Analysis of secondary structures using TMHMM revealed that GfsB and GfsC have putative transmembrane domains (amino acids 13 to 35 in GfsB and amino acids 23 to 42 in GfsC) at their N termini, suggesting that both GfsB and GfsC are type II membrane proteins and that both are localized to the Golgi apparatus, similarly to GfsA (3, 14). Both GfsB and GfsC have a conserved metal-binding DXD motif (amino acids 237 to 239 in GfsB and amino acids 240 to 242 in GfsC).

### Enzymatic function of GfsA, GfsB, and GfsC.

**FIG 1:**
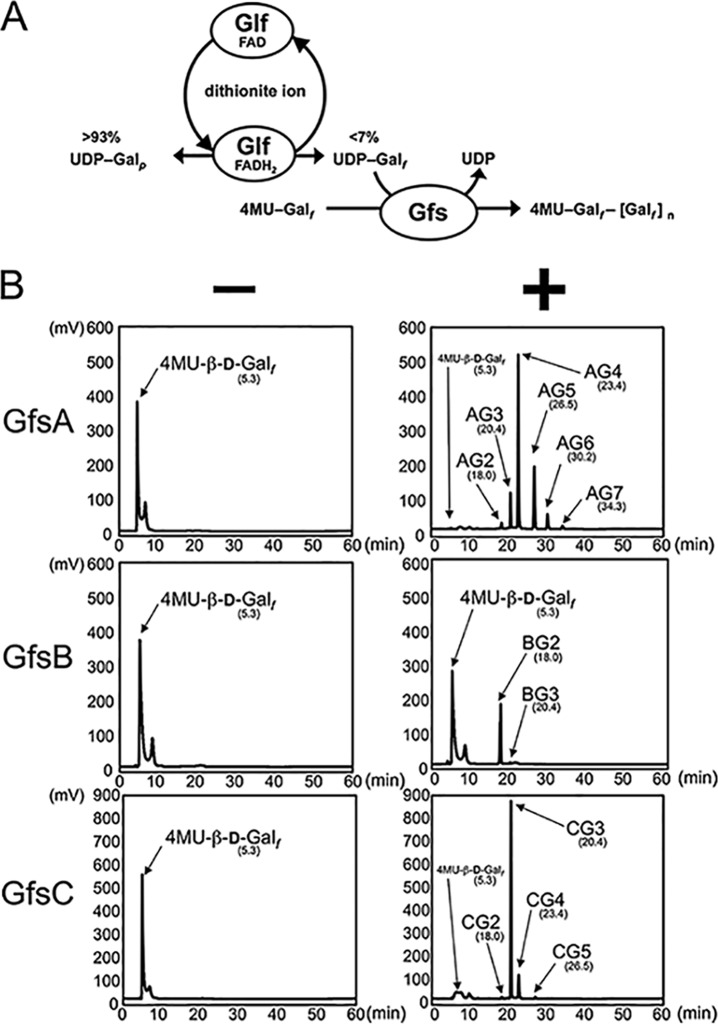
*In vitro* method for measuring galactofuranosyltransferase activity using a continuous reaction. (A) Schematic diagram of continuous reaction in galactofuranosyltransferase activity assay. Glf is UDP-galactopyranose (Gal*_p_*) mutase derived from E. coli to generate UDP-Gal*_f_* from UDP-Gal*_p_*. The enzymatic equilibrium of a reversible Glf enzyme is skewed >93% to UDP-Gal*_p_*. When a small amount of the UDP-Gal*_f_* generated is consumed by galactofuranosyltransferase, Glf reverts to UDP-Gal*_f_* to maintain equilibrium. Glf oxidizes FADH_2_ to FAD in the conversion from UDP-Gal*_p_* to UDP-Gal*_f_*. Therefore, reducing FAD to FADH_2_ is essential for a continuous reaction. Dithionite ions serve as driving forces for galactofuranosylation via the reduction of FADH_2_ from FAD within the continuous reaction. (B) Chromatograms of *in vitro* assay of galactofuranosyltransferase activity of GfsA, GfsB, and GfsC. Enzyme activities were assayed as described in Materials and Methods. Purified GfsA, GfsB, or GfsC (4.5 μg) was used as the enzyme. Chromatograms indicate typical results of the assay performed with and without GfsA, GfsB, or GfsC (right and left panels, respectively). The assays lacking GfsA, GfsB, or GfsC showed no novel peak generation (left panels), but in contrast, the fractions containing GfsA, GfsB, or GfsC did show new products (defined as AG2 to AG7 for GfsA, BG2 and BG3 for GfsB, and CG2 to CG5 for GfsC; right panels). Retention times were 18.0 min for AG2, BG2, and CG2; 20.4 min for AG3, BG3, and CG3; 23.4 min for AG4 and CG4; 26.5 min for AG5 and CG5; 30.2 for AG6; and 34.3 min for AG7. Numbers in parentheses indicate retention times of each enzymatic product. Gal*_f_*, galactofuranose; Gal*_p_*, galactopyranose; Glf, UDP-galactopyranose mutase from E. coli; FAD, flavin adenine dinucleotide; 4MU, 4-methylumbelliferyl.

We previously constructed an Escherichia coli strain expressing a recombinant GfsA protein. GfsC was successfully expressed as a soluble protein using a cold shock expression vector and GfsB as a soluble fused NusA protein by the use of an E. coli expression system. Recombinant 6×His-tagged GfsA, GfsB, and GfsC proteins were purified by Ni^+^ affinity chromatography and analyzed using SDS-PAGE (see Fig. S1 in the supplemental material). The NusA tag of GfsB was cleaved with a human rhinovirus (HRV) 3C protease and removed by the use of nickel-agarose. GfsA, GfsB, and GfsC were visualized as bands close to their predicted respective molecular weights of 57.9, 50.3, and 52.0 kDa. For the galactofuranosyltransferase assay, it was essential to use UDP-Gal*_f_* as a sugar donor. Because it was not commercially available, we biochemically synthesized UDP-Gal*_f_* using Glf, a UDP-galactopyranose (Gal*_p_*) mutase derived from E. coli, followed by purification by high-performance liquid chromatography (HPLC) (15, 16). This purified UDP-Gal*_f_* was also used for the assay in our previous study (4, 14). Because the enzymatic equilibrium of the reversible enzyme Glf was >93% of the mixture (16), generating significant UDP-Gal*_f_* levels was difficult. We therefore attempted to improve the galactofuranosyltransferase assay (Fig. 1A). When a small amount of generated UDP-Gal*_f_* was consumed by galactofuranosyltransferase, Glf regenerated UDP-Gal*_f_* to maintain the equilibrium (Fig. 1A). Glf oxidizes reduced flavin adenine dinucleotide (FADH_2_) to flavin adenine dinucleotide (FAD) by conversion of UDP-Gal*_p_* to UDP-Gal*_f_*. Therefore, reducing FAD to FADH_2_ was essential for a continuous reaction (Fig. 1A). Dithionite ions from sodium dithionite (SD) serve as driving forces for galactofuranosylation via the reduction of FAD to FADH_2_ within the reaction, which continues until UDP-Gal*_p_* is nearly depleted. On the basis of this principle, we developed a highly efficient assay for analysis of galactofuranosyltransferase activity using Glf and Gfs proteins (Fig. 1A). Chemically synthesized 4-methylumbelliferyl-β-d-galactofuranoside (4MU-β-d-*Galf*) or *p*-nitrophenyl-β-d-galactofuranoside (pNP-β-d-*Galf*) was used as an acceptor substrate (17, 18). The galactofuranosyltransferase reaction proceeded most efficiently when 4MU-Gal*_f_* and SD were used as the acceptor substrate and reducing agent, respectively (Fig. S2a and b). However, when commercially available pNP-β-d-*Galf* was used as an acceptor substrate, pNP-β-d-*Galf* was not detected via UV300 absorbance, suggesting that the nitrophenyl group was released by SD (Fig. S2d and e) (19). NADH can be used as a reducing agent for FAD instead of SD with either acceptor substrate. However, the efficiency was lower than that seen with SD (Fig. S2c and f).

10.1128/mSphere.00770-19.1 FIG S1 SDS-PAGE analysis of purified recombinant GfsA, GfsB, GfsC, and Glf proteins. Purified recombinant GfsA (5.0 μg), GfsB (3.0 μg), GfsC (5.0 μg), and Glf (5.0 μg) were separated by 5% to 20% SDS-PAGE and stained with Coomassie brilliant blue, revealing bands of approximately 57.9 kDa (GfsA), 50.3 kDa (GfsB), 52.0 kDa (GfsC), and 45.0 kDa (Glf). Download FIG S1, PDF file, 0.6 MB. Copyright © 2020 Chihara et al. 2020 Chihara et al. This content is distributed under the terms of the Creative Commons Attribution 4.0 International license.

10.1128/mSphere.00770-19.2 FIG S2 Effects of reducing agent and acceptor substrate on galactofuranosyltransferase. Chemically synthesized 4-methylumbelliferyl-β-d-galactofuranoside (4MU-β-d-Gal*_f_*) or *p*-nitrophenyl-β-d-galactofuranoside (pNP-β-d-Gal*_f_*) was used as an acceptor substrate. The standard galactofuranosyltransferase chromatogram assay was performed with purified GfsA protein (4.5 μg), purified 15.8 μg of Glf protein, 40 mM UDP-Gal*_p_*, 1 mM Mn^2+^, 1.5 mM 4MU-β-d-Gal*_f_*, and 40 mM sodium dithionite (SD) at 30°C for 16 h (a); with the 40 mM SD omitted from the standard assay (b); with 40 mM NADH added to the standard assay instead of 40 mM SD (c); with pNP-β-d-Gal*_f_* used as an acceptor substrate instead of 4MU-β-d-Gal*_f_* (d); with pNP-β-d-Gal*_f_* used as an acceptor substrate instead of 4MU-β-d-Gal*_f_* and 40 mM SD omitted from the standard assay (e); and with pNP-β-d-Gal*_f_* used as an acceptor substrate instead of 4MU-β-d-Gal*_f_* and 40 mM NADH added to the standard assay instead of 40 mM SD (f). Download FIG S2, PDF file, 0.1 MB. Copyright © 2020 Chihara et al. 2020 Chihara et al. This content is distributed under the terms of the Creative Commons Attribution 4.0 International license.

Fractions with GfsA exhibited six new peaks at 18.0, 20.4, 23.4, 26.5, 30.2, and 34.3 min (defined as AG2–AG7, respectively; Fig. 1B, upper panels). Fractions with GfsB had two new peaks at 18.0 and 20.4 min (BG2 and BG3, respectively; Fig. 1B, middle panels). Fractions with GfsC had four new peaks at 18.0, 20.4, 23.4, and 26.5 min (CG2 to CG5, respectively; Fig. 1B, bottom panels). All the enzyme-lacking fractions displayed no new peak generation (Fig. 1B, left panels). Table 1 shows the mass-to-charge ratios (*m*/*z*) of enzymatic products of GfsA, GfsB, and GfsC as identified by liquid chromatography-mass spectrometry (LC/MS). The value representing the differences corresponding to each peak was calculated as 162.1, indicating that a hexose molecule was continuously attached and that the mass corresponding to each peak was identical to the theoretical molecular mass of the molecule sequentially added to 4MU-β-d*-Galf* by Gal*_f_*.

**TABLE 1:** List of *m*/*z* ratios of enzymatic products of GfsA, GfsB, and GfsC identified by LC/MS^*a*^

Compound	Product name	Molecular mass (calculated)	Mass spectrum [M – H]^+^ (*m*/*z*)	Suggested structure
4MU-β-d-Gal		338.31	339.11	

GfsA	AG2	500.45	501.16	Gal*_f_*-β-(1→5)-Gal*_f_*-d-β-4MU
	AG3	662.59	663.21	Gal*_f_*-β-(1→5)-Gal*_f_*-β-(1→5)-Gal*_f_*-d-β-4MU
	AG4	824.73	825.27	Gal*_f_*-β-(1→5)-Gal*_f_*-β-(1→5)-Gal*_f_*-β-(1→5)-Gal*_f_*-d-β-4MU
	AG5	986.87	987.32	Gal*_f_*-β-(1→5)-Gal*_f_*-β-(1→5)-Gal*_f_*-β-(1→5)-Gal*_f_*-β-(1→5)-Gal*_f_*-d-β-4MU
	AG6	1,149.01	1,149.37	Gal*_f_*-β-(1→5)-Gal*_f_*-β-(1→5)-Gal*_f_*-β-(1→5)-Gal*_f_*-β-(1→5)-Gal*_f_*-β-(1→5)-Gal*_f_*-d-β-4MU
	AG7	1,311.15	1,311.42	Gal*_f_*-β-(1→5)-Gal*_f_*-β-(1→5)-Gal*_f_*-β-(1→5)-Gal*_f_*-β-(1→5)-Gal*_f_*-β-(1→5)-Gal*_f_*-β-(1→5)-Gal*_f_*-d-β-4MU

GfsB	BG2	500.45	501.16	Gal*_f_*-β-(1→5)-Gal*_f_*-d-β-4MU
	BG3	662.59	663.22	Gal*_f_*-β-(1→5)-Gal*_f_*-β-(1→5)-Gal*_f_*-d-β-4MU

GfsC	CG2	500.45	501.16	Gal*_f_*-β-(1→5)-Gal*_f_*-d-β-4MU
	CG3	662.59	663.21	Gal*_f_*-β-(1→5)-Gal*_f_*-β-(1→5)-Gal*_f_*-d-β-4MU
	CG4	824.73	825.27	Gal*_f_*-β-(1→5)-Gal*_f_*-β-(1→5)-Gal*_f_*-β-(1→5)-Gal*_f_*-d-β-4MU
	CG5	986.87	987.32	Gal*_f_*-β-(1→5)-Gal*_f_*-β-(1→5)-Gal*_f_*-β-(1→5)-Gal*_f_*-β-(1→5)-Gal*_f_*-d-β-4MU

a Products of GfsA were AG2, AG3, AG4, AG5, AG6, and AG7; those of GfsB were BG2 and BG3; and those of GfsC were CG2, CG3, CG4, and CG5. Mass-to-charge ratios (*m*/*z*) of the products were determined by LC/MS with positive-ion-mode electrospray ionization (ESI).

**FIG 2:**
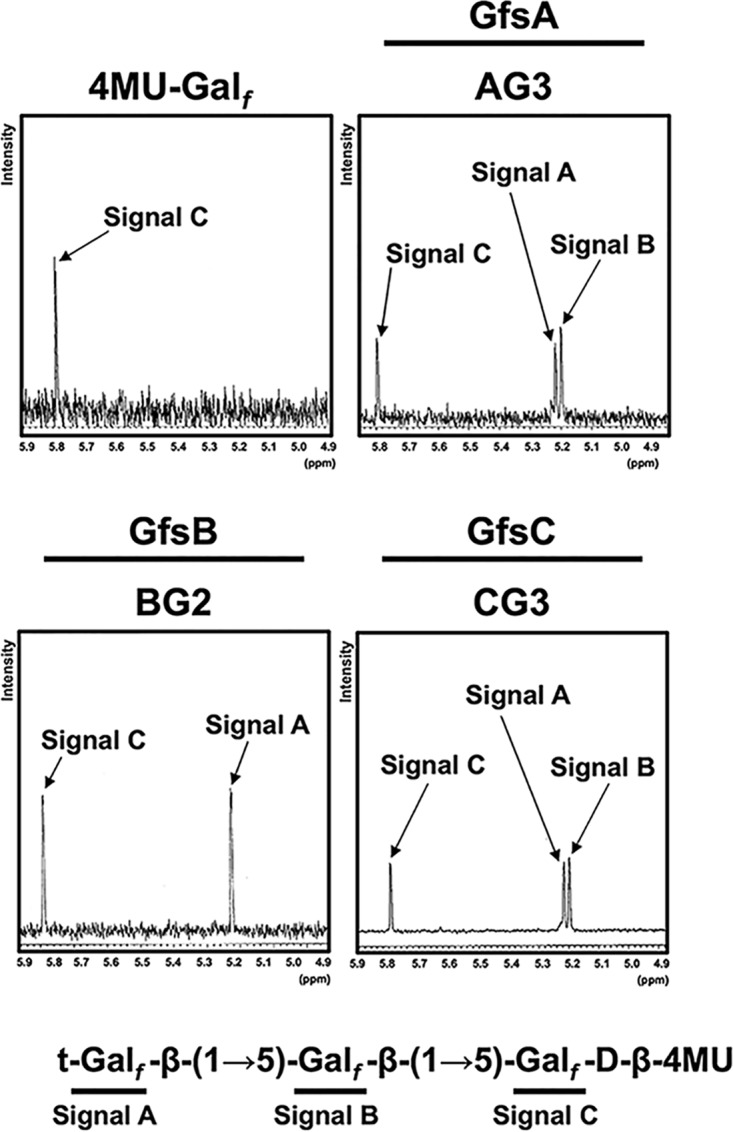
^1^H nuclear magnetic resonance (^1^H-NMR) analyses of enzymatic products of GfsA, GfsB, and GfsC using 4MU-β-d-*Galf* as an acceptor substrate. ^1^H-NMR charts are shown for 4MU-β-d*-Galf* (upper left), AG3 (upper right), BG2 (lower left), and CG3 (lower right). The 5.8-ppm signal was detected in the ^1^H-NMR chart for 4MU-β-d-*Galf*. The chemical shift values of BG2 of the H-1 position of the underlined Gal*_f_* residue in the Gal*_f_*-β-(1→5)-Gal*_f_*-β-4MU, and Gal*_f_*-β-(1→5)-Gal*_f_*-β-4MU structures are 5.22 and 5.79 ppm, respectively, according to previous reports (4, 6, 17, 18). The chemical shift values of AG3 and CG3 of the H-1 position of the underlined Gal*_f_* residue in the Gal*_f_*-β-(1→5)-Gal*_f_*-β-(1→5)-Gal*_f_*-β-4MU, Gal*_f_*-β-(1→5)-Gal*_f_*-β-(1→5)-Gal*_f_*-β-4MU, and Gal*_f_*-β-(1→5)-Gal*_f_*-β-(1→5)-Gal*_f_*-β-4MU structures are 5.22, 5.20 and 5.79 ppm, respectively, according to previous reports (4, 6, 17, 18).

**FIG 3:**
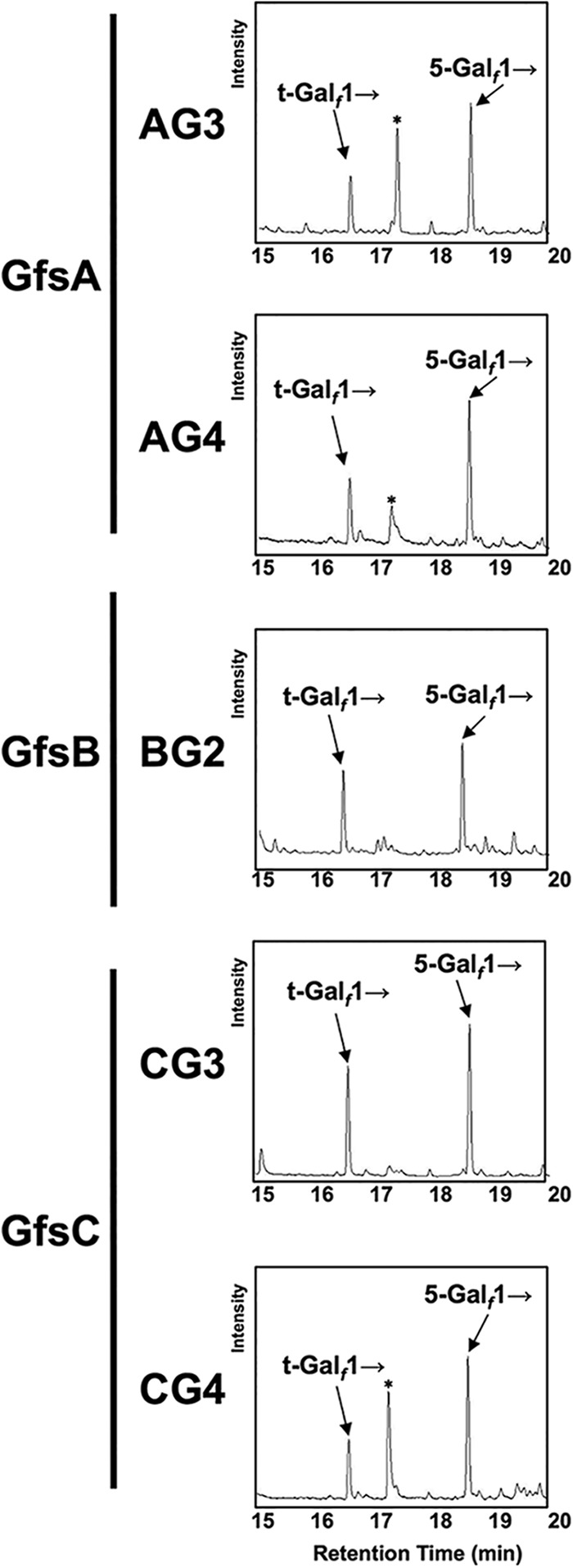
Methylation analyses of enzymatic products of GfsA, GfsB, and GfsC using 4MU-β-d-*Galf* as an acceptor substrate. Samples (500 μg) of AG3, AG4, BG2, CG3, and CG4 were analyzed. The retention times under these conditions used for analysis of t-Gal*_f_*→, 5-Gal*_f_*1→, and 6-Gal*_f_*1→ were 16.36, 18.40, and 19.56 min, respectively (4, 6, 20). The asterisk indicates an artificial peak that appearedfrequently.

To further determine chemical structure, we collected >1 mg of AG3, BG2, and CG3 by the use of HPLC and analyzed the sample using ^1^H nuclear magnetic resonance (^1^H-NMR) (Fig. 2) with 4MU-β-d-*Galf* as a control. The chemical shift values for the H-1 position of the Gal*_f_* residue in t-Gal*_f_*-β-(1→5)-Gal*_f_*-β-(1→5)-Gal*_f_*-d-β-4MU structures are 5.22 (signal A), 5.20 (signal B), and 5.79 (signal C) ppm from the nonreducing end, according to previous reports (4, 6). Signals for AG3, BG2, and CG3 were in agreement with the reported chemical shift values (Fig. 2). To obtain further evidence of glycosidic linkage, we collected 500 μg of AG3, AG4, BG2, CG3, and CG4 using HPLC and analyzed the sample using methylation analysis for each compound. The sample was methylated and then hydrolyzed and was subsequently analyzed by gas chromatography-mass spectrometry (GC-MS) (Fig. 3). The retention times for t-Gal*_f_*→, 5-Gal*_f_*1→, and 6-Gal*_f_*1→ were 16.36, 18.40, and 19.56 min, respectively, under these analysis conditions (6, 20). AG3, AG4, BG2, CG3, and CG4 displayed a peak at 16.36 min (Fig. 3), indicating the presence of terminal Gal*_f_* residues. In addition, AG3, AG4, BG2, CG3, and CG4 had a peak at 18.40 but not 19.56 min (Fig. 3), indicating that the added Gal*_f_* residue was attached to the C-5 position of the first Gal*_f_* residue. We found that the area ratios from the results of methylation analysis of CG3 and CG4 were not 1:2 and 1:3, suggesting that 5-Gal*_f_*1→ had been partially lost in the process of complex methylation analysis. These results indicate that GfsB and GfsC are also β-(1→5)-galactofuranosyltransferases and that GfsA, GfsB, and GfsC could not transfer a Gal*_f_* residue to the C-6 position, in contrast to GlfT2, the bacterial β-(1→5)-/β-(1→6)-galactofuranosyltransferase (21, 22).

### Role of GfsB and GfsC in GM biosynthesis.

**FIG 4:**
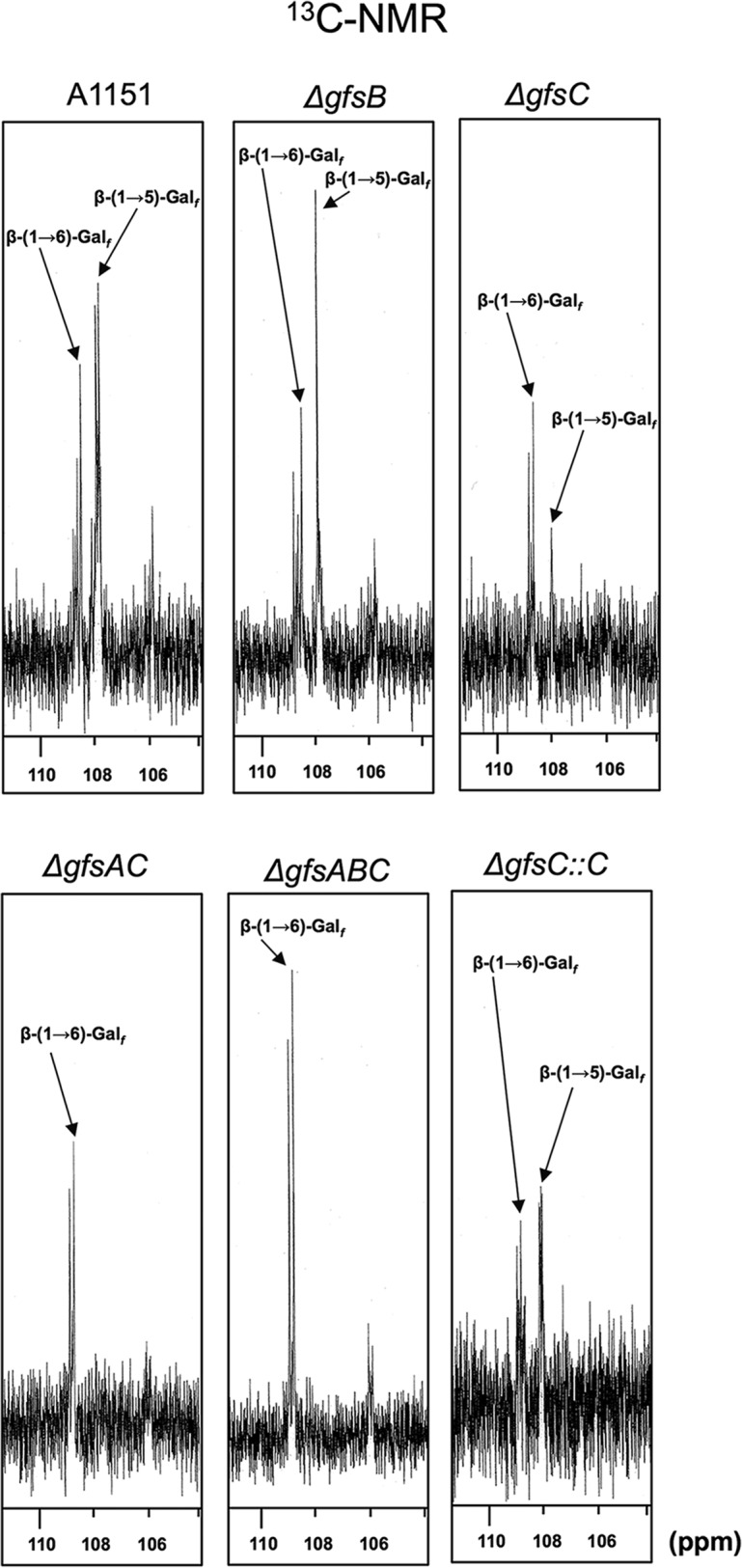
^13^C-NMR analyses of purified FTGM+OMGM fractions from the A1151, Δ*gfsB*, Δ*gfsC*, Δ*gfsAB*, Δ*gfsABC*, and Δ*gfsC*::*C* strains. The signals at 107.87 ppm and 108.70 ppm represent a C-1 chemical shift of the underlined Gal*_f_* residue in the Gal*_f_*-β-(1→5)-Gal*_f_*-β-(1→5)-Gal*_f_* [β-(1→5)-Gal*_f_*] and Gal*_f_*-β-(1→5)-Gal*_f_*-β-(1→6)-Gal*_f_* [β-(1→6)-Gal*_f_*] structures, respectively (6, 20). The carbon chemical shifts were referenced relative to internal acetone at 31.07 ppm. OMGM, *O*-mannose-type galactomannan; FTGM, fungal-type galactomannan; FTGM+OMGM, total GM (FTGM plus OMGM).

**FIG 5:**
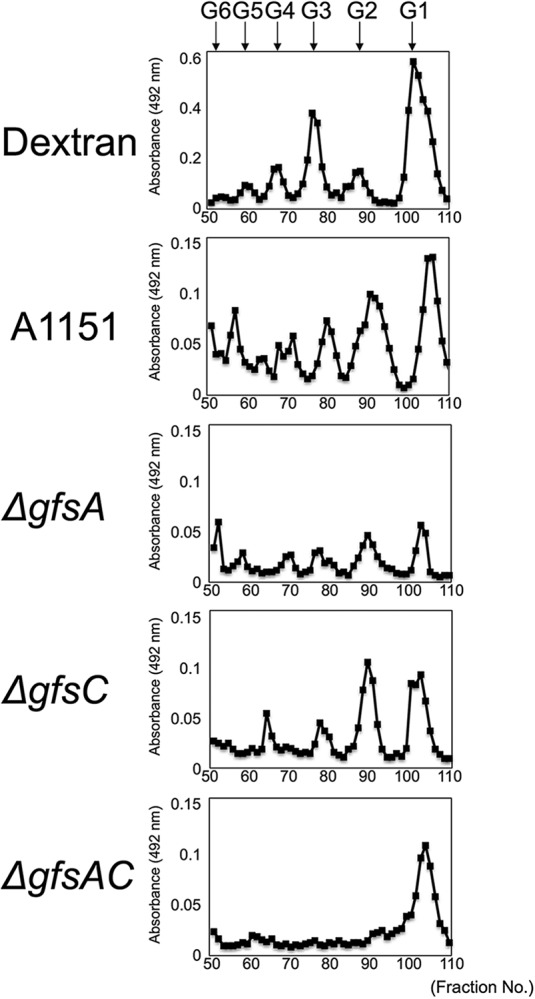
Analysis of galactofuran side chain length of fungal-type galactomannan. Galactofuran side chains were prepared and separated by gel filtration chromatography. FTGM+OMGM fractions were treated with 0.15 M trifluoroacetic acid at 100°C for 15 min. Gel filtration chromatography using a Bio-Gel P-2 (2-by-90-cm) column and distilled water (dH_2_O) as the eluent was applied to the resultant samples. The partial acid hydrolysis product of dextran was used as a molecular weight marker. The eluted sugar was detected using the phenol-sulfuric acid method. G1, glucose; G2, maltose; G3, maltotriose; G4, maltotetraose; G5, maltopentaose; G6, maltohexaose; G7, maltoheptaose.

To clarify the function of the *gfs* family *in vivo*, we constructed Δ*gfsB*, Δ*gfsC*, Δ*gfsC*::*C*, Δ*gfsAC*, and Δ*gfsABC* strains (Fig. S3, S4, and S5). To identify the effect of gene disruption on the structure of GMs, those extracted from the mycelia of A. fumigatus strains were purified by cetyl trimethyl ammonium bromide precipitation with boric acid buffer. The GMs designated FTGM+OMGM contain both FTGM and OMGM (4); these were analyzed by ^13^C-NMR spectroscopy (Fig. 4). Signals at 107.87 ppm and 108.70 ppm of the ^13^C-NMR spectra represent the C-1 positions of the underlined Gal*_f_* residue within the structure of -Gal*_f_*-β-(1→5)-Gal*_f_*-[1→ (β-(1→5)-Gal*_f_*] and -Gal*_f_*-β-(1→6)-Gal*_f_*-[1→ (β-1→6)-Gal*_f_*], respectively, in accordance with previous reports (6, 23). The signal intensity of β-(1→5)-Gal*_f_* was higher than that of β-(1→6)-Gal*_f_* in the ^13^C-NMR chart of the A1151-FTGM+OMGM strain. Within the Δ*gfsB*-FTGM+OMGM strain there was little difference from the A1151-FTGM+OMGM strain (Fig. 4). In contrast, the intensity of signal of β-(1→5)-Gal*_f_* was inversed in the ^13^C-NMR chart of the Δ*gfsC*-FTGM+OMGM strain, indicating that the amount of β-(1→5)-Gal*_f_* was decreased in the FTGM+OMGM fraction of Δ*gfsC* strains (Fig. 4). The signal intensity of β-(1→5)-Gal*_f_* was recovered in the ^13^C-NMR chart of the Δ*gfsC*::*C*-FTGM+OMGM strain (Fig. 4). Interestingly, no signals of β-(1→5)-Gal*_f_* in the ^13^C-NMR chart of the Δ*gfsAC-* and Δ*gfsABC*-FTGM+OMGM strain were detected, indicating that β-(1→5)-Gal*_f_* disappeared within the FTGM+OMGM fractions of the Δ*gfsAC* and Δ*gfsABC* strains. GC-MS analyses of *O*-methylalditol acetates derived from methylation analyses of FTGM+OMGMs were performed for the A1151, Δ*gfsB*, Δ*gfsC*, Δ*gfsAC*, Δ*gfsABC*, and Δ*gfsC*::*C* strains (Table 2). The ratio of the 5-*O*-substituted Gal*_f_* residue (5-Gal*_f_*1→) of the Δ*gfsC* strain (2.16% ± 0.19%) was lower than that of the A1151 strain (16.31% ± 0.84%); however, the ratio of 5-Gal*_f_*1→ of the Δ*gfsB* strain (15.37% ± 0.71%) was comparable with that of the A1151 strain (Table 2). Interestingly, no signals for the 5-Gal*_f_*1→ of the Δ*gfsAC* strain or the Δ*gfsABC* strain were detected within these FTGM+OMGM fractions (Table 2). These results clearly indicate that β-(1→5)-galactofuranosyl residues disappeared within both the Δ*gfsAC* strain and the Δ*gfsABC* strain. Next, the FTGM galactofuran side chain was prepared and separated by gel filtration chromatography to analyze its length (Fig. 5). FTGM+OMGM fractions were treated with 0.15 M trifluoroacetic acid at 100°C for 15 min. The resultant samples were applied to gel filtration chromatography to separate the obtained galactofuran side chain (Fig. 5). In the Δ*gfsC* strain, each β-(1→5)-galactofuranosyl chain containing mainly two residues was shorter than that in the parent strain (Fig. 5), but the length of each β-(1→5)-galactofuranosyl chain did not appear to be affected by the deletion of *gfsA*. The peak of each β-(1→5)-galactofuranosyl chain was lower in the Δ*gfsA* strain than in the parent and Δ*gfsC* strains (Fig. 5). These results imply that GfsA and GfsC have different roles in galactofuran side chain biosynthesis. Most importantly, only monosaccharide was detected in the Δ*gfsAC* strain fraction, indicating that elongation by β-(1→5)-galactofuranosyl residues had not occurred (Fig. 5). These observations clearly indicate that all β-(1→5)-galactofuranosyl residues of FTGM and OMGM in A. fumigatus are biosynthesized by GfsA and GfsC.

**TABLE 2:** GC-MS analysis of *O*-methylalditol acetates derived from methylation analyses of galactomannans

*O*-Methylalditol acetate	Sugar linkage	Residue ratio for indicated strain^*a*^							
		A. fumigatus A1151	A. fumigatus Δ*gfsA*	A. fumigatus Δ*gfsB*	A. fumigatus Δ*gfsC*	A. fumigatus Δ*gfsAC*	A. fumigatus Δ*gfsABC*	A. fumigatus Δ*gfsA*::*A*	A. fumigatus Δ*gfsC*::*C*
2,3,4,6-Me4-Man	tMan*_p_*1→	17.01 ± 1.05	28.40 ± 9.78	21.19 ± 3.48	22.89 ± 2.96	26.50 ± 1.00	23.13 ± 5.45	19.12 ± 1.75	16.32 ± 1.83
3,4,6-Me3-Man	2-Man*_p_*1→	29.80 ± 2.28	21.34 ± 3.78	27.89 ± 2.34	26.69 ± 1.25	25.23 ± 5.11	25.04 ± 2.39	15.99 ± 2.46	21.35 ± 3.15
2,3,4-Me3-Man	6-Man*_p_*1→	12.95 ± 1.66	11.67 ± 0.53	12.76 ± 1.54	16.42 ± 1.09	16.80 ± 1.87	16.39 ± 1.24	14.96 ± 2.03	8.50 ± 0.74
3,4-Me2-Man	2,6-Man*_p_*1	8.36 ± 0.86	8.65 ± 1.20	7.14 ± 1.33	8.25 ± 1.28	7.62 ± 0.92	8.99 ± 1.26	9.47 ± 1.47	4.06 ± 0.40
2,3,5,6-Me4-Gal	tGal*_f_*1→	13.76 ± 3.33	14.94 ± 1.60	14.29 ± 2.04	19.97 ± 1.27	20.94 ± 2.35	22.77 ± 1.09	20.01 ± 0.94	26.86 ± 1.51
2,3,6-Me3-Gal	5-Gal*_f_*1→	16.31 ± 0.84	10.75 ± 4.32	15.37 ± 0.71	2.16 ± 0.19	N. D.	N. D.	16.67 ± 1.27	20.18 ± 2.12
2,3,5-Me3-Gal	6-Gal*_f_*1→	1.81 ± 0.70	4.21 ± 0.61	1.34 ± 0.24	3.62 ± 0.44	2.91 ± 0.68	3.68 ± 0.51	3.78 ± 1.37	2.73 ± 0.87

a The data representing the Δ*gfsA* and Δ*gfsA*::*A* strains were adopted from Katafuchi et al. (4).

10.1128/mSphere.00770-19.3 FIG S3 Construction of the Δ*gfsB*, Δ*gfsC* (*ptrA*), and Δ*gfsC* (*AnpyrG*) strains. (a and b) Chromosomal maps of the Δ*gfsB* strain (a) and the Δ*gfsC* strain (b) and the primers used for confirmation. The positions of the primers are indicated by arrows. (c and d) Electrophoretic analyses of products amplified by PCR using primer pairs AFUB_070620-1/ptrA-R (5′ region) and ptrA-F/AFUB_070620-4 (3′ region) for the Δ*gfsB* strain (c) and primer pairs AFUB_067290-1/pyrG-R (5′ region) and pyrG-F/AFUB_067290-4 (3′ region) for the Δ*gfsC* strain (d). M, DNA size markers (Gene Ladder Wide 2; Nippon Gene, Tokyo, Japan). Download FIG S3, PDF file, 0.1 MB. Copyright © 2020 Chihara et al. 2020 Chihara et al. This content is distributed under the terms of the Creative Commons Attribution 4.0 International license.

10.1128/mSphere.00770-19.4 FIG S4 Construction of the Δ*gfsC* complementary strains Δ*gfsC*::*C*. (a) Schematic representation of Δ*gfsC* complementation with *gfsC*. *gfsC* (P), *gfsC* promoter; *gfsC* (T), *gfsC* terminator; *gfsC*, open reading frame of *gfsC*. The positions of the primers are indicated by arrows. (b) Confirmation of correct recombination of *gfsC* using PCR analysis. Results of electrophoretic analysis of products amplified by PCR are shown. M, DNA size markers (Gene Ladder Wide 2); lane 1, DNA fragment (2.6 kbp) amplified using PCR and the primers AfgfsC-complement-7 and ptrA-R; lane 2, DNA fragment (1.1 kb) amplified using PCR and the primers ptrA-F and AfgfsC-complement-8. Download FIG S4, PDF file, 0.08 MB. Copyright © 2020 Chihara et al. 2020 Chihara et al. This content is distributed under the terms of the Creative Commons Attribution 4.0 International license.

10.1128/mSphere.00770-19.5 FIG S5 Construction of the Δ*gfsAC* and Δ*gfsABC* strains. (a and b) Chromosomal maps of the Δ*gfsAC* strain (a) and Δ*gfsABC* strain (b) and primers used for confirmation. The positions of the primers are indicated by arrows. (c and d) Electrophoretic analyses of products amplified by PCR using primer pairs AFUB_096220-1/pyrG-R (5′ region of *gfsA*), pyrG-F/AFUB_096220-4 (3′ region of *gfsA*), AFUB_067290-1/ptrA-R (5′ region of *gfsC*), and ptrA-F/AFUB_067290-4 (3′ region of *gfsC*) for the Δ*gfsAC* strain (c) and primer pairs AFUB_096220-1/pyrG-R (5′ region of *gfsA*), pyrG-F/AFUB_096220-4 (3′ region of *gfsA*), AFUB_070620-1/hygB-R (5′ region of *gfsB*), hygB-F/AFUB_070620-4 (3′ region of *gfsB*), AFUB_067290-1/ptrA-R (5′ region of *gfsC*), and ptrA-F/AFUB_067290-4 (3′ region of *gfsC*) for the Δ*gfsABC* strain (d). M, DNA size markers (Gene Ladder Wide 2). Download FIG S5, PDF file, 0.2 MB. Copyright © 2020 Chihara et al. 2020 Chihara et al. This content is distributed under the terms of the Creative Commons Attribution 4.0 International license.

### Phenotypic analyses of disruptant *gfs* family genes.

**FIG 6:**
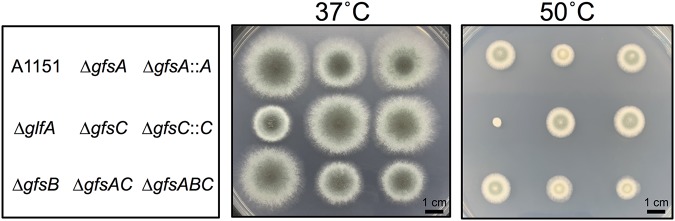
Colony phenotype comparison of A1151, Δ*gfsA*, Δ*gfsB*, Δ*gfsC*, Δ*gfsAB*, Δ*gfsABC*, *gfsA*::*A* and Δ*gfsC*::*C* strains. Strain colony images are shown for the A1151, Δ*gfsA*, Δ*gfsB*, Δ*gfsC*, Δ*gfsAB*, Δ*gfsABC*, *gfsA*::*A*, and Δ*gfsC*::*C* strains. Conidia were incubated on minimal medium at 37°C (left) or 50°C (right) for 3 days.

The colony phenotypes of disruptant strains were observed following 3 days of growth at 37°C/50°C on minimal medium (MM) (Fig. 6). The colony growth rates of the disruptant strains are shown in Table 3. The colony growth rate of the Δ*gfsA* strain decreased to 85.2% of that of the A1151 strain at 37°C (Table 3). In contrast, the colony growth rate percentages of the Δ*gfsB* and Δ*gfsC* strains were comparable with that of the A1151 strain at 37°C (Table 3). The growth rates of the Δ*gfsAC* and Δ*gfsABC* strains were reduced to 68.4% and 67.8% at 37°C and to 86.4% and 84.0% at 50°C, respectively (Table 3). Quantifying the number of formed conidia at 37°C, the proportion of the Δ*gfsA* strain decreased to 50.9% compared with that of the A1151 strain. The conidiation efficiencies of the Δ*gfsAC* and Δ*gfsABC* strains were reduced to approximately 32.1% and 25.4% of that of the A1151 strain (Table 4). In contrast, the conidiation efficiency of the Δ*gfsB* and Δ*gfsC* strains did not obviously decrease (Table 4). These results are consistent with the findings showing that GfsA and GfsC play important roles in the biosynthesis of β-(1→5)-galactofuranosyl chains whereas GfsB has little function *in vivo*. These results indicate that the β-(1→5)-galactofuranosyl residues play an important role in conidium formation and hyphal growth.

**TABLE 3:** Colony growth rate of the A1151, Δ*gfsA*, Δ*gfsB*, Δ*gfsC*, Δ*gfsAC*, Δ*gfsABC*, Δ*gfsA*::*A*, andΔ*gfsC*::*C* strains

Temp (°C)	Growth rate, mm^2^/h (% of strain A1151 growth rate)							
	A. fumigatus A1151	A. fumigatus Δ*gfsA*	A. fumigatus Δ*gfsB*	A. fumigatus Δ*gfsC*	A. fumigatus Δ*gfsAC*	A. fumigatus Δ*gfsABC*	A. fumigatus Δ*gfsA*::*A*	A. fumigatus Δ*gfsC*::*C*
37	0.75 ± 0.06 (100)	0.63 ± 0.04 (85.2)	0.73 ± 0.10 (98.6)	0.76 ± 0.08 (102.0)	0.51 ± 0.03 (68.4)	0.50 ± 0.05 (67.8)	0.78 ± 0.04 (104.1)	0.76 ± 0.06 (101.9)
50	0.30 ± 0.04 (100)	0.24 ± 0.03 (81.2)	0.30 ± 0.03 (100.5)	0.27 ± 0.11 (90.9)	0.26 ± 0.03 (86.4)	0.25 ± 0.03 (84.0)	0.31 ± 0.03 (103.2)	0.37 ± 0.03 (124.3)

**TABLE 4:** Number of formed conidia of the A1151, Δ*gfsA*, Δ*gfsB*, Δ*gfsC*, Δ*gfsAC*, Δ*gfsABC*, Δ*gfsA*::*A*, and Δ*gfsC*::*C* strains

Strain name	No. of formed conidia/mm^2^	% of formed conidia compared to WT strain
A. fumigatus A1151	3.1 × 10^5^ ± 9.6 × 10^4^	100
A. fumigatus Δ*AfgfsA*	1.6 × 10^5^ ± 9.1 × 10^3^	50.9
A. fumigatus Δ*AfgfsB*	3.0 × 10^5^ ± 5.2 × 10^4^	95.9
A. fumigatus Δ*AfgfsC*	2.8 × 10^5^ ± 3.0 × 10^4^	90.9
A. fumigatus Δ*AfgfsAC*	1.0 × 10^5^ ± 5.7 × 10^3^	32.1
A. fumigatus Δ*AfgfsABC*	7.9 × 10^4^ ± 3.5 × 10^3^	25.4
A. fumigatus Δ*AfgfsA*::*A*	2.6 × 10^5^ ± 3.5 × 10^4^	82.6
A. fumigatus Δ*AfgfsC*::*C*	3.8 × 10^5^ ± 3.4 × 10^4^	120.3

**FIG 7:**
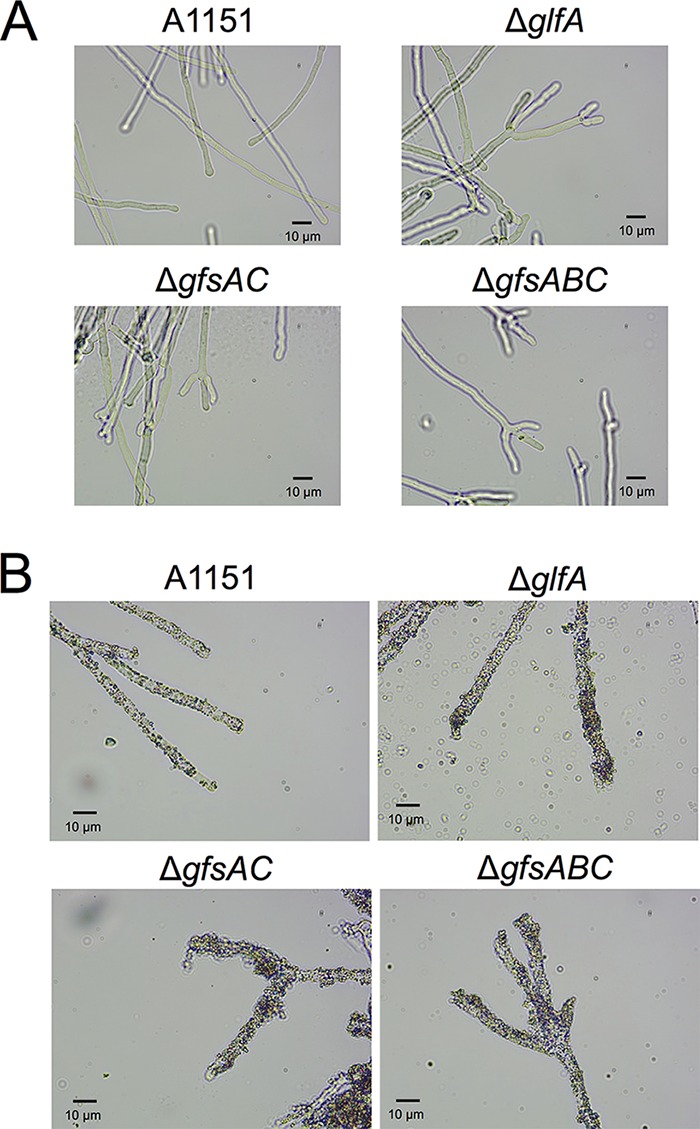
Morphology of the A1151, Δ*glfA*, Δ*gfsAC*, and Δ*gfsABC* strains. (A) Morphology of hyphae of the A1151, Δ*glfA*, Δ*gfsAC*, and Δ*gfsABC* strains. (B) Hydrophobicity of the hyphae of the A1151, Δ*glfA*, Δ*gfsAC*, and Δ*gfsABC* strains. Hydrophobicity is indicated by adherence of latex beads to the hyphae.

It was reported previously that branching of hyphae was increasing within the Δ*glfA* strain (24). Therefore, we observed hyphae in Δ*gfsAC* and Δ*gfsABC* strains to determine whether abnormal branching of hyphae was also occurring in these strains (Fig. 7A). We did observe abnormal branching of hyphae at a high frequency, indicating that deficiency of β-(1→5)-galactofuranosyl residues causes an increase of abnormal branching of hyphae. Reportedly, lack of the Gal*_f_*-containing sugar chains from the cell causes increased cell surface hydrophobicity (24). To confirm increasing cell surface hydrophobicity in the Δ*gfsAC* and Δ*gfsABC* strains, we determined whether the amount of adherence of latex beads to the mycelium was increased as observed within the Δ*glfA* strain; this adherence was clearly increased (Fig. 7B), indicating that β-(1→5)-galactofuranosyl residues are involved in cell surface hydrophobicity in A. fumigatus.

### Sensitivity to antifungal agents and virulence of β-(1→5)-galactofuranosyl residue-deficient strains.

**FIG 8:**
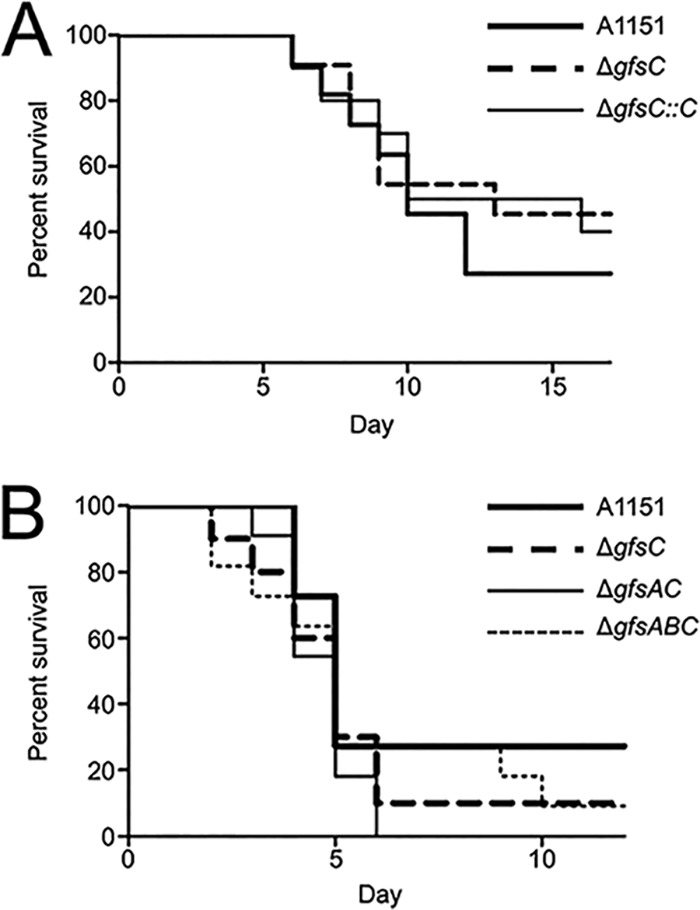
β-(1→5)-Galactofuranosyl residues are dispensable for virulence in a mouse model of invasive pulmonary aspergillosis. (A) Infection with A1151, Δ*gfsC*, and Δ*gfsC*::*C* strains. Outbred ICR mice (male; 5 weeks of age; *n* = 11) were immunocompromised via intraperitoneal injection of cyclophosphamide (200 mg/kg of body weight) at days −4, −2, 2, and 5. Cortisone acetate was also administered subcutaneously at a concentration of 200 mg per kg on day −1. Mice were infected intratracheally with 3.0 × 10^5^ conidia in a volume of 30 μl for each strain (including the A1151, Δ*gfsC*, and Δ*gfsC*::*C* strains) on day 0. (B) Mouse infection with A1151, Δ*gfsC*, Δ*gfsAC*, and Δ*gfsABC* strains. Outbred ICR mice (male; 5 weeks of age; *n* = 10 to 11) were immunocompromised via intraperitoneal injection of cyclophosphamide (200 mg/kg mouse) at days −4, −2, 2, and 3. Cortisone acetate was also administered subcutaneously at a concentration of 200 mg per kg on day −1. Mice were infected intratracheally with 3.0 × 10^5^ conidia in a volume of 30 μl for each strain (A1151, Δ*gfsC*, Δ*gfsAC*, and Δ*gfsABC* strains) on day 0.

Next, we tested the sensitivity of the A1151, Δ*gfsC*, Δ*gfsAC*, and Δ*gfsABC* strains to the widely used clinical antifungal agents micafungin (MCFG), caspofungin (CPFG), amphotericin B (AMPH-B), flucytosine (5-FC), fluconazole (FLCZ), itraconazole (ITCZ), voriconazole (VRCZ), and miconazole (MCZ) (Table 5). The sensitivities of the mutants to antifungal agents were almost identical to those of strain A1151. The Δ*gfsABC* strain exhibited only slightly greater sensitivity to AMPH-B and MCZ than the A1151 strain (Table 5). We also examined the role of β-(1→5)-galactofuranosyl residues in pathogenesis using a murine infection model (Fig. 8). First, the levels of virulence of the A1151, Δ*gfsC*, and Δ*gfsC*::*C* strains were tested within immunocompromised mice. Survival rates did not differ between the A1151, Δ*gfsC*, and Δ*gfsC*::*C* infections (Fig. 8A). The levels of virulence of the A1151, Δ*gfsC*, Δ*gfsAC*, and Δ*gfsABC* strains were also tested (Fig. 8B). In the aspergillosis model, the virulence levels of the Δ*gfsAC* and Δ*gfsABC* strains were comparable with that of the A1151 strain (Fig. 8B), indicating that a lack of β-(1→5)-galactofuranosyl residues did not influence the survival rates of immunosuppressed mice.

**TABLE 5:** Sensitivity of the A1151, Δ*gfsC*, Δ*gfsAC*, and Δ*gfsABC* strains to antifungal agents^*a*^

Strain	MIC (μg/ml)							
	MCFG	CPFG	AMPH-B	5-FC	FLCZ	ITCZ	VRCZ	MCZ
A. fumigatus A1151	0.015	0.25	1	>64	>64	0.5	0.5	2
A. fumigatus Δ*gfsC*	0.015	0.25	1	>64	>64	0.5	0.5	1–2
A. fumigatus Δ*gfsAC*	0.015	0.25	1	>64	>64	0.25–0.5	0.5–1	1–2
A. fumigatus Δ*gfsABC*	0.015	0.25	0.5	>64	>64	0.5	0.5	1

a MCFG, micafungin; CPFG, caspofungin; AMPH-B, amphotericin B; 5-FC, flucytosine; FLCZ, fluconazole; ITCZ, itraconazole; VRCZ, voriconazole; MCZ, miconazole.

## DISCUSSION

**FIG 9:**
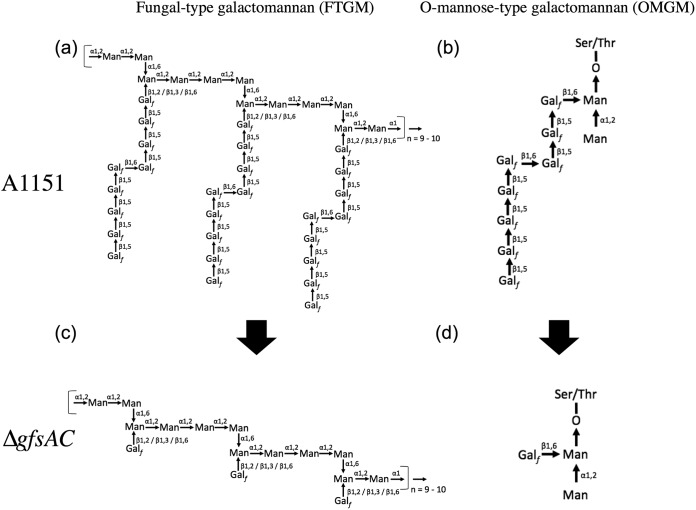
Proposed GM structures in Δ*gfsAC* strain. (a) Typical structure of FTGM. (b) OMGM in A. fumigatus proposed by Kudoh et al. (6). (c) Proposed structure of FTGM. (d) OMGM in the Δ*gfsAC* strain.

Our previous characterization showed that GfsA is the β-galactofuranoside β-(1→5)-galactofutanosyltransferase (4). However, the β-(1→5)-galactofuranosyl oligomer synthesized with GfsA could only be confirmed to generate up to 3 sugars in the previous reaction system due to a lack of commercially available UDP-Gal*_f_* (4). In this study, we showed that GfsA could synthesize β-(1→5)-galactofuranosyl oligomers up to lengths of 7 monosaccharides *in vitro* (Fig. 1, upper panels). In addition, we showed that GfsB and GfsC also could transfer β-Gal*_f_* to the 5 position of the hydroxy group of the terminal β-galactofuranosyl residue at up to lengths of 3 and 5 monosaccharides *in vitro*, respectively (Fig. 1, middle and bottom panels). AG4, BG2, and CG3 accumulated within the assays of GfsA, GfsB, and GfsC *in vitro*, respectively (Fig. 1). The β-(1→5)-galactofuranosyl chain structures synthesized by GfsA and/or GfsC *in vitro* were identical to those of β-(1→5)-linked trigalactofuranoside and β-(1→5)-linked tetragalactofuranoside (Fig. 9a and b), which are *in vivo* structures proposed previously by Kudoh et al. (6). From the data representing the structural analysis of GM extracted from the Δ*gfsAC* strain, we clearly demonstrated that β-(1→5)-galactofuranosyl residues disappeared in the Δ*gfsAC* strain. These data indicate that GfsA and GfsC have redundant enzymatic functions *in vitro* and *in vivo* and that these enzymes are responsible for the biosynthesis of all β-(1→5)-galactofuranosyl chains (Fig. 4 and 5; see also Table 2). We subsequently proposed the structures of the fungal-type and *O*-mannose-type GMs in the Δ*gfsAC* strain (Fig. 9c and d). However, the observations in this study also imply that GfsA and GfsC have different roles in the biosynthesis of FTGM and/or OMGM (Fig. 5 and 6; see also Table 2). As reported by Kudoh et al., A. fumigatus changes the length of galactofuran side chains in response to external conditions (6). In addition, biological plasticity is also induced due to genetic defects. Clarifying the complex mechanism is extremely meaningful and interesting. However, the functional differences between GfsA and GfsC are unknown, necessitating further detailed analysis.

One problematic issue for assaying galactofuranosyltransferases is that the sugar donor UDP-Gal*_f_* is not commercially available. Errey et al. previously described relatively easily synthesizing UDP-Gal*_f_* using flexible enzymatic and chemoenzymatic approaches (25). However, obtaining and retaining the chemically unstable UDP-Gal*_f_* remain complicated (26). We thus attempted to establish a galactofuranosyltransferase assay using a continuous reaction of sugar-nucleotide conversion and sugar transfer with UDP-galactopyranose mutase and galactofuranosyltransferase. Rose et al. previously performed an assay to detect galactofuranosyltransferase activity via a continuous reaction using NADH for the reduction of FAD (27). In our hands, galactofuranosylation proceeded even when NADH was used instead of SD but was more efficient with SD than with NADH (see Fig. S2a and c in the supplemental material). This established method could measure galactofuranosyltransferase activity without the addition of UDP-Gal*_f_*. In addition, since a sufficient amount of purified product was separated and purified, this is advantageous for structural analysis of the enzymatic product (Fig. 2 and 3). This method will likely be useful for functional analysis of other galactofuranosyltransferases.

The phenotypic abnormalities in the growth of the Δ*gfsA* strain were less severe than those of the Δ*gfsAC* and Δ*gfsABC* strains (Fig. 6; see also Tables 3 and 4). Deletion of *gfsB* and *gfsC* did not result in any growth defect of A. fumigatus (Fig. 6; see also Tables 3 and 4). Very recently, similar results have been observed within disrupted strains of *gfs* family genes in A. niger (28), suggesting the existence of common functional relationships of the *gfs* family proteins for A. niger and A. fumigatus. We clarified that the phenotypic abnormalities occurring in the Δ*gfsAC* strain were due to defects in β-(1→5)-galactofuranosyl residues via analysis of the sugar chain structure of the Δ*gfsAC* strain.

Several mutants in which galactofuranosyl sugar chains are absent have been reported previously in A. fumigatus (24, 29, 30); whole galactofuranosyl sugar chains are absent within Δ*glfA* and Δ*glfB* strains. These absent Gal*_f_* residues caused decreased growth rates, abnormal branching of hyphae, decreased thickness of cell walls, increased susceptibility to several antifungal agents, and increased adhesive phenotype compared with the parental strain (24, 29, 30). The phenotypes of the Δ*gfsAC* and Δ*gfsABC* strains were similar in some aspects but not identical to that of the Δ*glfA* strain. The latter showed stronger inhibition of hyphal growth and conidium formation than the Δ*gfsAC* and Δ*gfsABC* strains (Fig. 6). This was because galactofuranosyl residues are β-(1→5)-linked, β-(1→2)-linked, β-(1→3)-linked, and β-(1→6)-linked sugars (5, 6, 10). These Gal*_f_* residues, except β-(1→5)-galactofuranosyl, are found in glycosyl phosphoinositolceramides (GIPC), FTGM, OMGM, and N-glycans (5, 6, 10, 31–33) and might be involved in biological events. Only the β-(1→5)-galactofuranosyl residues disappeared in the Δ*gfsAC* and Δ*gfsABC* strains; thus, it seems reasonable that those strains exhibit less influence than the Δ*glfA* strain, wherein the entirety of the Gal*_f_*-containing sugar chain is lost. However, the results showed that abnormal branching of the hyphae and cell surface hydrophobicity were not significantly different between the Δ*gfsAC*, Δ*gfsABC*, and Δ*glfA* strains (Fig. 7), indicating that the functions of GM β-(1→5)-galactofuranosyl residues are heavily involved in normal polarity of the hyphae and in cell surface hydrophobicity.

The presence of the β-(1→6)-galactofuranosyl moiety has been reported in the galactofuran side chain of FTGM and OMGM in A. fumigatus. Therefore, we predicted that if the β-(1→5)-galactofuranosyl residues disappeared, so would the β-(1→6)-galactofuranosyl residues. Upon the disappearance of the β-(1→5)-galactofuranosyl residues, however, the β-(1→6)-galactofuranosyl residues remained detectable within the ^13^C-NMR of GMs from the Δ*gfsAC* strain (Fig. 4). This strongly suggests the existence of a β-(1→6)-galactofuranosyl oligomer and/or polymer other than the β-(1→6)-galactofuranosyl moiety of the FTGM galactofuran side chain. A β-(1→6)-galactofuranosyl polymer was previously found in *Fusarium* sp. but not in A. fumigatus (34–36).

In the mouse infection model of invasive aspergillosis, the absence of GM β-(1→5)-galactofuranosyl residues did not result in significant differences in virulence for the A1151, Δ*gfsAC* and Δ*gfsABC* strains. These findings were consistent with Lamarre’s findings that disruption of *glfA* has no effect on virulence (24). In contrast, Schmalhorst et al. previously reported that disruption of *glfA* resulted in attenuated virulence in a mouse model of invasive aspergillosis (29). Recently, using a zebrafish embryo model, Koch et al. showed that the survival rate of Δ*glfA* strain (Schmalhorst’s strain) decreased slightly more gradually than that of the wild-type strains (37). They explained that the attenuated pathogenicity of the Δ*glfA* strain might be caused by decreased germination rate or hyphal growth rate (37). These differences in virulence might be due to the differing genetic backgrounds of the strains used or to the differing protocols of the pathogenicity tests, necessitating further detailed analysis to understand the involvement of β-(1→5)-galactofuranosyl sugar chains in pathogenicity.

This study broadened our understanding of the biosynthesis of β-(1→5)-galactofuranosyl residues in A. fumigatus and of their important role in cell wall formation. However, β-(1→6)-galactofuranosyltransferases that transfer β-galactofuranoses to galactofuranosyl residues have not been identified in A. fumigatus. Additionally, no β-(1→2)- and β-(1→3)-/β-(1→6)-galactofuranosyltransferases transferring β-galactofuranose to mannosyl residues have been identified. Our findings regarding the biosynthesis of β-(1→5)-galactofuranosyl residue provide important novel insights into the formation of the complex cell wall structure and the virulence of the subphylum Pezizomycotina. Future studies will be needed to identify other galactofuranosyltransferases and to clarify the individual functions of each Gal*_f_*-containing oligosaccharide.

## MATERIALS AND METHODS

### Microorganisms and growth conditions.

The A. fumigatus strains used in this study are listed in Table S1 in the supplemental material. A. fumigatus A1160 and A1151 were obtained from the Fungal Genetics Stock Center (FGSC) (38). Strains were grown on minimal medium (MM). Standard transformation procedures for *Aspergillus* strains were used. Plasmids were amplified in E. coli DH5α. E. coli strain Rosetta-gami B (DE3) (purchased from Merck Millipore of Germany) was used for protein expression. Colony growth rates were measured as described previously (14).

10.1128/mSphere.00770-19.6 TABLE S1 *Aspergillus* strains used in this study. Download Table S1, PDF file, 0.06 MB. Copyright © 2020 Chihara et al. 2020 Chihara et al. This content is distributed under the terms of the Creative Commons Attribution 4.0 International license.

### Construction of GfsB and GfsC expression vector.

All PCRs were performed using Prime STAR GXL DNA polymerase (TaKaRa Bio, Otsu, Japan). pCold II (TaKaRa Bio) and pET50b-Amp plasmids were used for protein expression in E. coli. pET50b-Amp is a plasmid that was constructed by replacing the kanamycin resistance gene of pET50b with an ampicillin resistance gene as follows. The DNA region of pET50b except for the kanamycin resistance gene was amplified by PCR using plasmid pET50b as a template for primer pair pET50b-Amp-F and pET50b-Amp-R. The ampicillin resistance gene was amplified by PCR using plasmid pET15b as a template for primer pair Amp-gene-F and Amp-gene-R. The obtained DNA fragments were ligated using an In-Fusion HD cloning kit (TaKaRa Bio) to yield pET50b-Amp. Plasmids useful for expression of *gfsB* and *gfsC* were constructed as follows. Total RNA was extracted from A. fumigatus A1160 strain mycelia grown in MM for 18 h using TRIzol reagent (Thermo Fisher Scientific, MA, USA) according to the manufacturer’s instructions. Single-stranded DNA was synthesized by the use of Moloney murine leukemia virus (M-MLV) reverse transcriptase (Nippon Gene, Tokyo, Japan) and oligo(dT)-18 primers. *gfsB* and *gfsC* were amplified using PCR with single-stranded DNA as a template for primer pair pET50b-AfGfsB-F and pET50b-AfGfsB-R for *gfsB* and primer pair pCold2-AfGfsC-F and pCold2-AfGfsC-R for *gfsC*. The amplified fragments were inserted into the NdeI site of pCold II to yield pCold2-AfGfsC and into the SmaI site of pET50b-Amp to yield pET50b-Amp-AfGfsB using an In-Fusion HD cloning kit. The constructed plasmids were transformed into Rosetta-gami B (DE3) cells.

### Protein purification, quantification, and electrophoresis.

GfsA protein was expressed in Rosetta-gami B (DE3) cells harboring pET15b-AfGfsA plasmids (4). Protein expression and purification were performed for GfsA and GfsB as described previously (4). Rosetta-gami B (DE3) cells harboring pCold2-AfGfsC plasmids were used for protein expression of GfsC, which was performed according to the manufacturer’s protocol for the pCold DNA cold shock expression system. The NusA tag of GfsB was cleaved with a HRV 3C protease (TaKaRa Bio) at 4°C and removed by the use of nickel-agarose. Protein concentrations were determined using a Qubit protein assay kit (Thermo Fisher Scientific), and purified proteins were analyzed by SDS-PAGE to assess purity and molecular weight. Glf protein was obtained with the ASKA clone as previously described (4, 39). Purified Glf was visualized as a band close to the predicted molecular weight of 45.0 kDa (see Fig. S1 in the supplemental material).

### Synthesis of *p*-nitrophenyl β-d-galactofuranoside (pNP-Gal*_f_*) and 4-methylumbelliferyl β-d-galactofuranoside (4MU-Gal*_f_*).

*para*-Nitrophenyl β-d-galactofuranoside (pNP-Gal*_f_*) was chemically synthesized as described previously (18, 40) or purchased (Toronto Research Chemicals, Toronto, Canada). 4-Methylumbelliferyl β-d-galactofuranoside (4MU-Gal*_f_*) was chemically synthesized (41, 42) as follows. 4-Methylumbelliferon (50.0 mmol) and BF_3_·Et_2_O (50.0 mmol) were added to a solution of perbenzoylated galactofuranose (10.0 mmol) with 4A molecular sieves in CH_3_CN (50 ml) at 0°C (41). The reaction mixture was stirred at 0°C for 1 h followed by 23°C for 24 h. Next, the mixture was filtered through a Celite pad and the residue was diluted with EtOAc, washed with saturated aqueous (sat. aq.) NaHCO_3_ solution and brine, dried over MgSO_4_, and concentrated *in vacuo* to dryness, producing a mixture of 4-methylumbelliferyl 2,3,4,6-tetra-*O*-benzoyl-β-d-galactofuranoside. A 28% aq. NH_3_ solution was added to the aforementioned mixture in CH_3_OH at 0°C, and the resulting solution was stirred at that temperature for 1 h and then at 23°C for 24 h. The reaction solution was concentrated. The target material was purified by silica-gel column chromatography (CHCl_3_:CH_3_OH, 4/1) to give 4-methylumbelliferyl β-d-galactofuranoside (4MU-Gal*_f_*) as a yellow solid (1.80 mmol).

### Continuous enzymatic reaction assay.

Standard assays were performed with 1.5 mM 4MU-β-d-*Galf* acceptor substrate, 40 mM UDP-galactopyranose, purified Glf protein (UDP-galactopyranose mutase from Escherichia coli; 15.8 μg), 40 mM sodium dithionite (SD), and purified GfsA (4.5 μg), GfsB (4.5 μg), or GfsC (4.5 μg) protein in a total reaction volume of 20 μl. The mixtures were incubated at 30°C for 16 h, and the reaction was stopped by the application of heat (99°C) for 5 min. The supernatants were analyzed by HPLC with an amino column (Shodex Asahipak NH2P-50 4E; Showa Denko, Tokyo, Japan) (250 by 4.6 mm) as previously described (4). 4-Methylumbelliferyl and *p*-nitrophenyl derivatives were detected by 300 nm of absorbance. The mass spectra of the enzymatic products of GfsA, GfsB, and GfsC were determined using an Exactive Plus Orbitrap mass spectrometer (Thermo Fisher Scientific).

### Construction of Δ*gfsB* and Δ*gfsC* gene disruption strains.

A. fumigatus A1151/A1160 was used as parental strain (Table S1). *gfsB* was disrupted in the A1151 strain by *ptrA* insertion; *gfsC* was also disrupted in the A1160 strain by *AnpyrG* insertion. DNA fragments for gene disruption were constructed using a “double-joint” PCR method as described previously (43). The 5′- and 3′-flanking regions (approximately 1.0 to 1.1 kb each) of each gene were PCR amplified from genomic DNA with the following primer pairs (Table S2): primer pair AFUB_070620-1/AFUB_070620-2 and primer pair AFUB_070620-3/AFUB_070620-4 for *gfsB* disruption and primer pair AFUB_067290-1/AFUB_067290-2 and primer pair AFUB_067290-3/AFUB_067290-4 for *gfsC*. *ptrA* and *AnpyrG*, used as selective markers, were amplified using plasmids pPTR-I (TaKaRa Bio) and pSH1 (14) as the templates, respectively, and primer pair ptrA-5/ptrA-6 or primer pair pyrG-5/pyrG-6. The three amplified fragments were purified and mixed, and a second PCR was performed without specific primers to assemble each fragment, as the overhanging chimeric extensions act as primers. A third PCR was performed with nested primer pair AFUB_070620-7/AFUB_070620-8 for *gfsB* or AFUB_067290-7/AFUB_067290-8 for *gfsC* and the products of the second PCR as a template to generate the final deletion construct. The amplified final deletion constructs were purified with a Fast Gene gel/PCR extraction kit (Nippon Gene) and used directly for transformation. Transformants were grown on MM plates containing 0.6 M KCl as an osmotic stabilizer under appropriate selection conditions, and single colonies were isolated twice before further analysis. Disruption of target genes was confirmed by PCR with the following primer pairs: primer pairs AFUB_070620-1/ptrA-R and ptrA-F/AFUB_070620-4 for *gfsB* and primer pairs AFUB_067290-1/ptrA-R and ptrA-F/AFUB_067290-4 or AFUB_067290-1/pyrG-R and pyrG-F/AFUB_067290-4 for *gfsC* (Fig. S3).

10.1128/mSphere.00770-19.7 TABLE S2 Oligonucleotides used in this study. Download Table S2, PDF file, 0.06 MB. Copyright © 2020 Chihara et al. 2020 Chihara et al. This content is distributed under the terms of the Creative Commons Attribution 4.0 International license.

### Construction of the complementary strain of Δ*gfsC* using *gfsC*.

The A. fumigatus Δ*gfsC* strain was used as the parental strain (Table S1). The relevant region of *gfsC* was PCR amplified from genomic DNA using primer pair AfgfsC-complement-1/AfgfsC-complement-2 (Table S2). The relevant region of *AnpyrG* was PCR amplified from pSH1 using primer pair AfgfsC-complement-3/AfgfsC-complement-4 (Table S2). *ptrA* genes, used as selective markers, were amplified using plasmid pPTR-I as a template and primer pair ptrA-5/ptrA-6. The three amplified fragments were purified and mixed, and a second PCR was performed. A third PCR was performed using the nested primer pair AfgfsA-complement-7/AfgfsA-complement-8 and the products of the second PCR as a template to generate the final DNA construct. Correct replacement of the DNA fragments for gene complementation was confirmed by PCR using primer pairs AfgfsA-complement-1/ptrA-R and ptrA-F/AfgfsA-complement-4 (Fig. S4).

### Construction of double and triple gene disruption strains.

A. fumigatus Δ*gfsA* was used as a parental strain (Table S1) to construct double and triple gene disruption strains. Genes were disrupted in A. fumigatus by *ptrA* or *hph* insertion; *gfsC* was disrupted in strain Δ*gfsA* by *ptrA* insertion to construct strain Δ*gfsAC*. Next, *gfsB* was disrupted in strain Δ*gfsAC* by *hph* insertion to construct strain Δ*gfsABC*. Primer pairs AFUB_067290-1/AFUB_067290-2(*gfsC*::*ptrA*), AFUB_067290-3(*gfsC*::*ptrA*)/AFUB_067290-4, ptrA-5/ptrA-6, and AFUB_067290-7/AFUB_067290-8 were used to construct a deletion cassette for Δ*gfsC*. Primer pairs AFUB_070620-1/AFUB_070620-2(*gfsB*::*hph*), AFUB_070620-3(*gfsB*::*hph*)/AFUB_070620-4, hph-5/hph-6, and AFUB_070620-7/AFUB_070620-8 were used to construct a deletion cassette for Δ*gfsB*. *hph* was amplified by PCR using pAN7-1 as a template (44) and primers hph-5 and hph-6 (Table S2). Target gene disruption was confirmed using PCR with primer pairs AFUB_096220-1/pyrG-R and pyrG-F/AFUB_096220-4 for *gfsA*, AFUB_070620-1/hph-R and hph-F/AFUB_070620-4 for *gfsB*, and AFUB_067290-1/ptrA-R and ptrA-F/AFUB_067290-4 for *gfsC* (Fig. S5).

### Methylation analysis and nuclear magnetic resonance (NMR) spectroscopy.

GM was prepared using Lloyd’s method with a slight modification as described previously (4, 14, 45). Briefly, the cell extract was dissolved in 3% cetyl trimethylammonium bromide (CTAB) solution, and then the GM fraction was recovered as a precipitate in the presence of 1% borate at pH 9.5 with a few drops of 1 M NaOH. The obtained fraction was washed with 75% ethanol, dialyzed in water, and then lyophilized. Glycosidic linkage was analyzed using a previously described method (4, 6). NMR experiments were performed as previously described (4, 6, 14). Proton and carbon chemical shifts were referenced relative to internal acetone at δ 2.225 and 31.07 ppm, respectively.

### Analysis of conidiation efficiency and surface adhesion.

Conidiation efficiency was analyzed as described previously (14). Hyphal surface adhesion assay was performed as described previously with sight modifications (24, 46). The 0.5-μm-diameter polystyrene beads (Sigma) were diluted 1:100 in sterile phosphate-buffered saline (PBS). Mycelia were grown for 18 h at 37°C with shaking at 127 rpm in liquid MM, harvested into PBS-containing polystyrene beads for 1 h, and then washed five times with PBS. Mycelium images were collected using a microscope equipped with a digital camera.

### Drug susceptibility testing and mouse model of pulmonary aspergillosis.

Drug susceptibility assays were performed in triplicate as described previously (12, 47, 48). The mouse model of pulmonary aspergillosis was generated per a previously described method (49) with slight modifications. In each experiment, A1151, Δ*gfsC*, and Δ*gfsC*::*C* strains or A1151, Δ*gfsC*, Δ*gfsAC*, and Δ*gfsABC* strains were used to infect immunosuppressed mice (10 or 11 mice per group). Outbreed male ICR mice were housed in sterile cages (5 or 6 per cage) with sterile bedding and provided with sterile feed and drinking water containing 300 μg/ml tetracycline hydrochloride to prevent bacterial infection. Mice were immunosuppressed with cyclophosphamide (200 mg per kg of body weight), which was intraperitoneally administered on days −4, −2, 2, and 5, or −4, −2, and 3 (day 0: infection). Cortisone acetate (200 mg per kg of body weight) was injected on day −1 for immunosuppression. Mice were infected by intratracheal instillation of 3 × 10^5^ conidia in a mixture containing 30 μl of PBS. Mice were weighed and visually inspected every 24 h from the day of infection. On recording 30% body weight reduction, the mouse was regarded as moribund and euthanized. The Prism statistical analysis package was used for statistical analysis and survival curve drawing (GraphPad Software Inc., CA, USA).

### Ethics statement.

The institutional animal care and use committee of Chiba University approved the animal experiments (permit no. DOU28-376 and DOU29-215). All efforts were made to minimize suffering in strict accordance with the principles outlined by the Guideline for Proper Conduct of Animal Experiments.
