# Supplementary figures and images for: Biosynthesis of β-(1→5)-Galactofuranosyl Chains of Fungal-Type and O-Mannose-Type Galactomannans within the Invasive Pathogen Aspergillus fumigatus
Source: mSphere. 2020 Jan 15;5(1):e00770-19. doi: 10.1128/mSphere.00770-19 (PMC6968653; doi:10.1128/mSphere.00770-19)

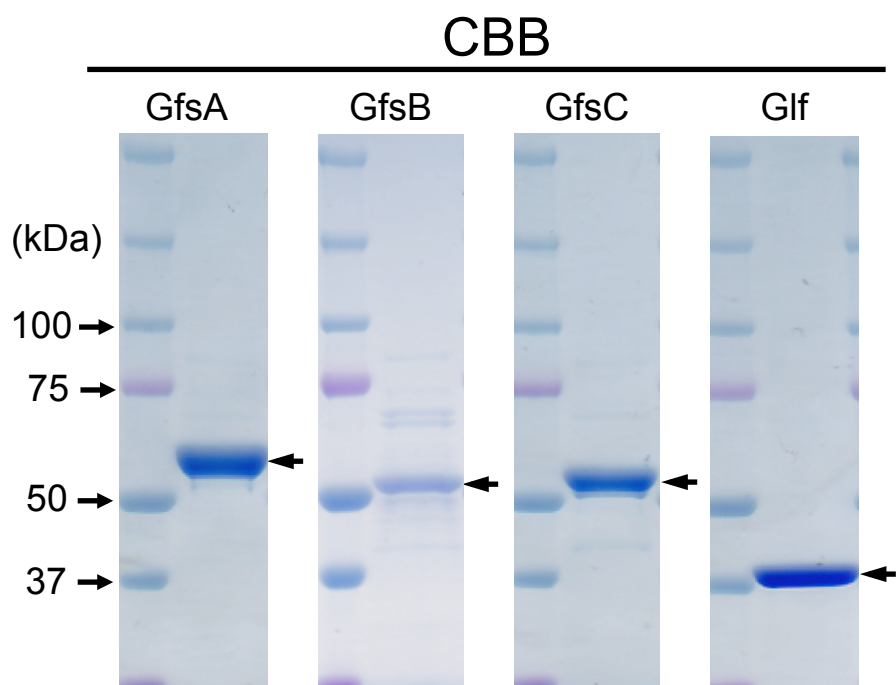

Fig. S1

Supplement: FIG S1 [file mSphere.00770-19-sf001.pdf]

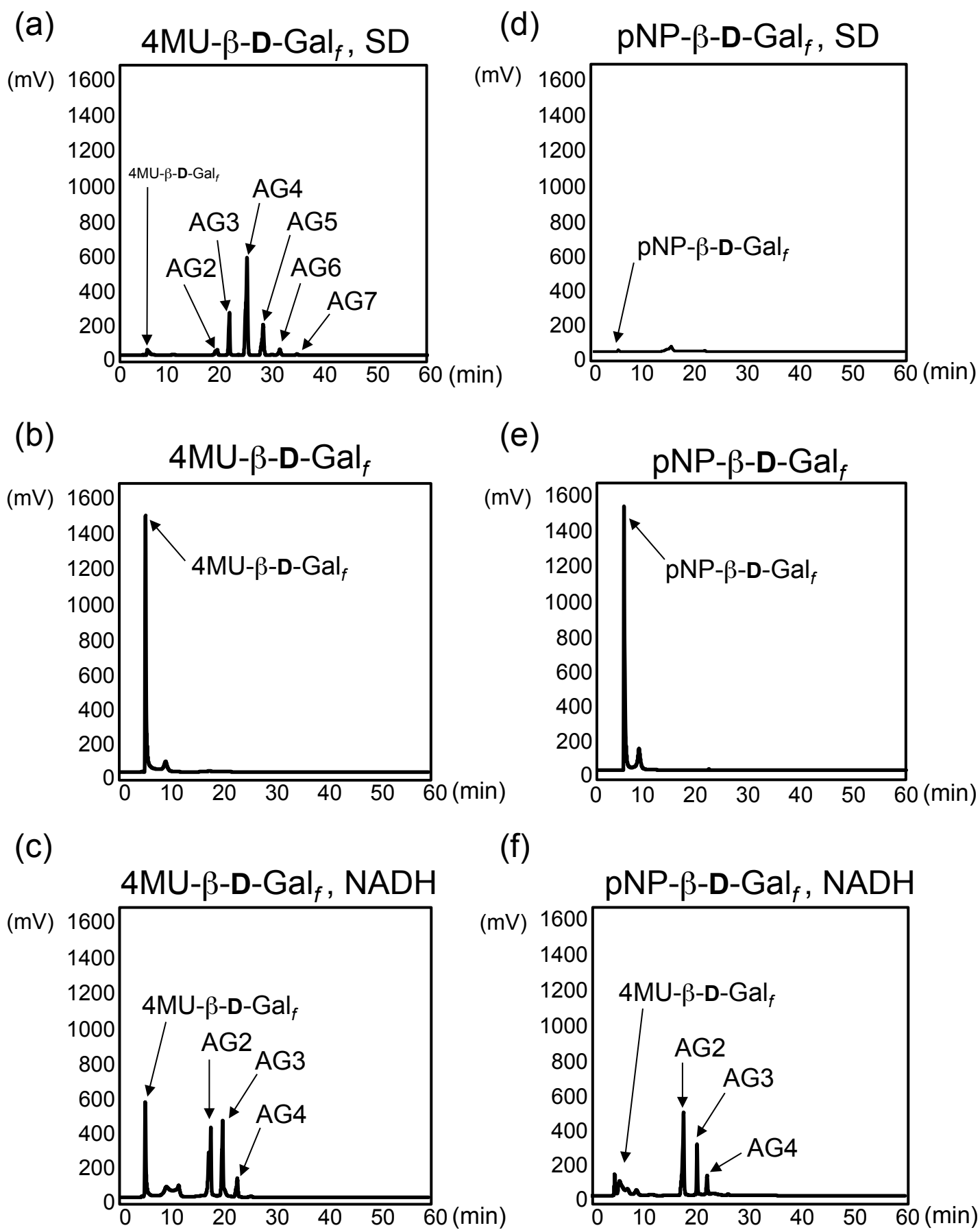

Fig. S2

Supplement: FIG S2 [file mSphere.00770-19-sf002.pdf]

(a) *ΔgfsB*

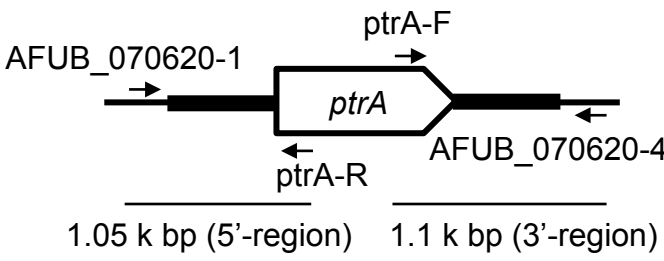

(c)

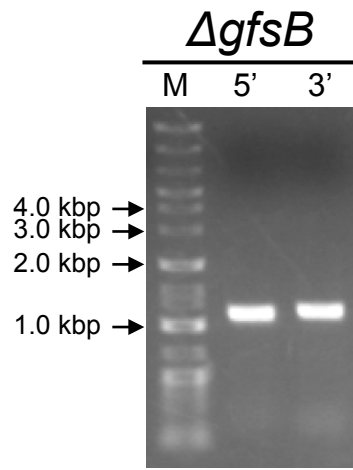

(b) *ΔgfsC*

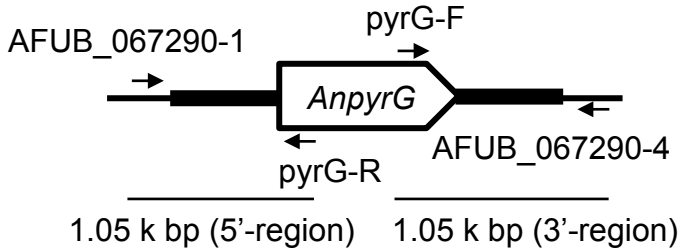

(d)

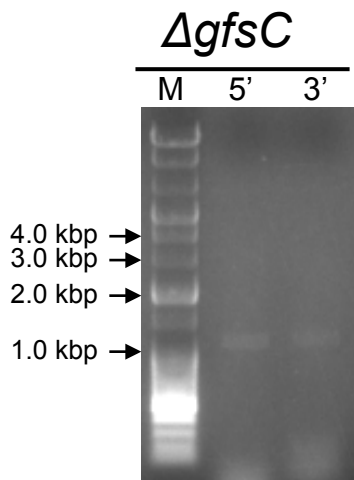

Fig. S3

Supplement: FIG S3 [file mSphere.00770-19-sf003.pdf]

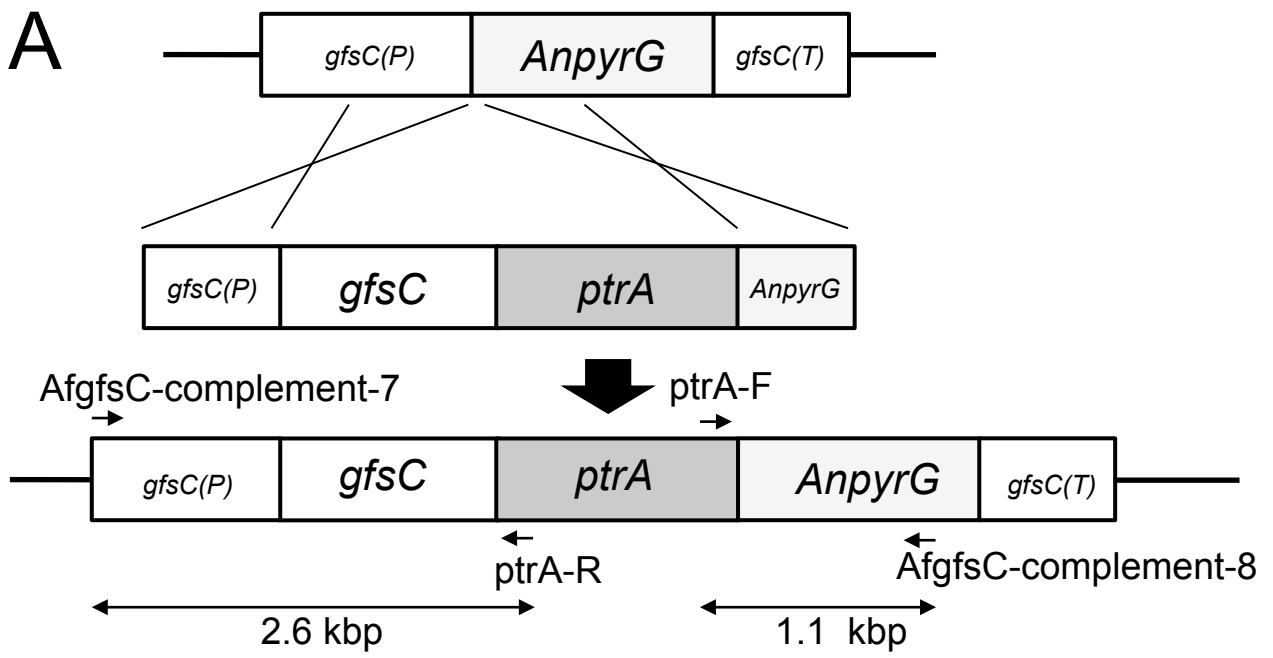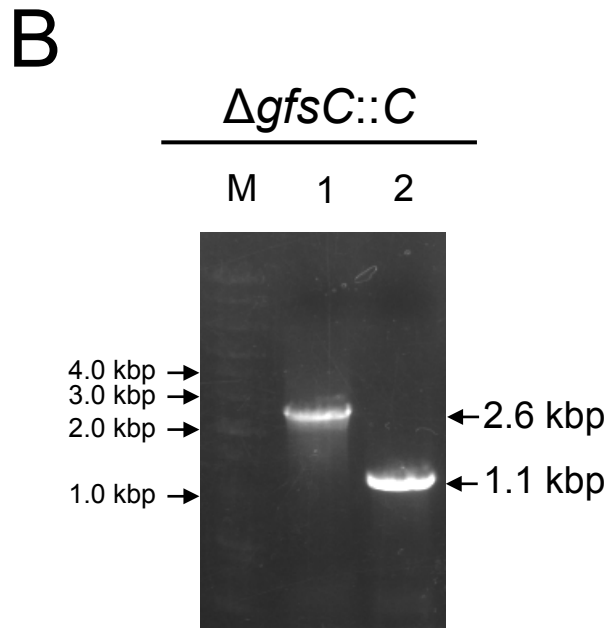

Fig. S4

Supplement: FIG S4 [file mSphere.00770-19-sf004.pdf]

(a) *ΔgfsAC*

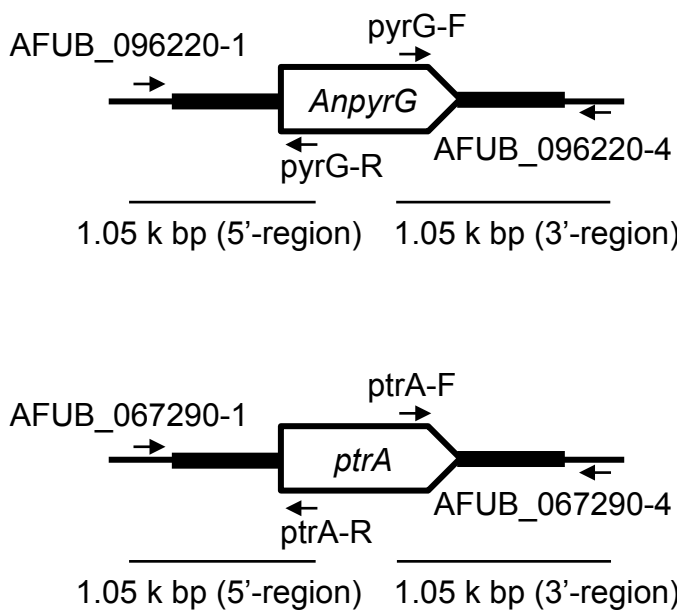

(c) *ΔgfsAC*

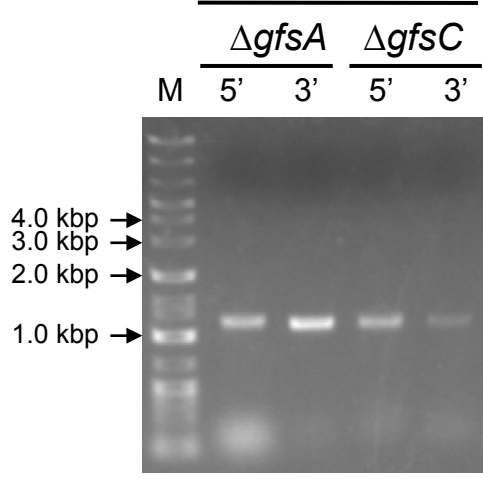

(b) *ΔgfsABC*

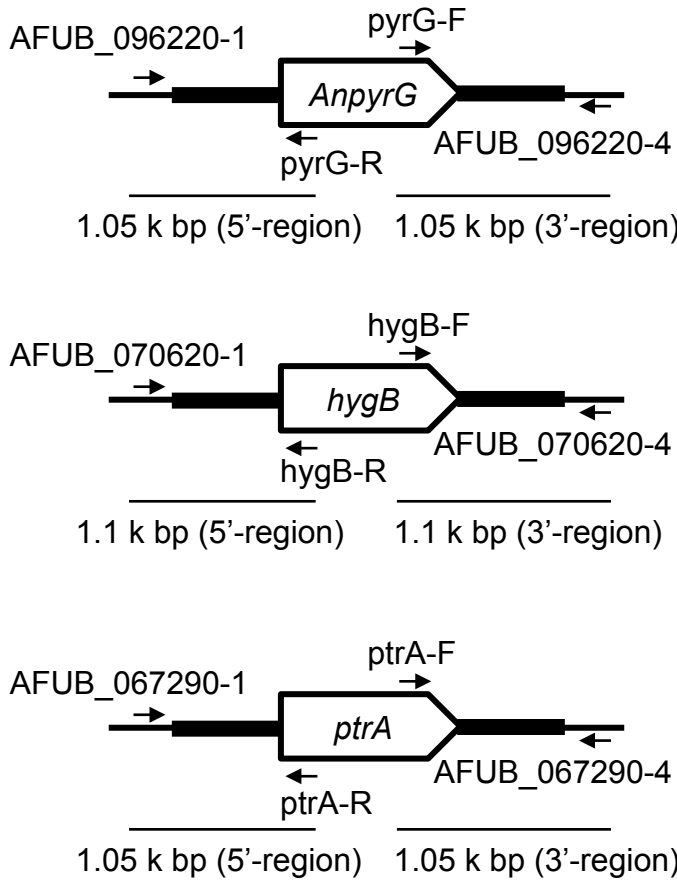

(d) *ΔgfsABC*

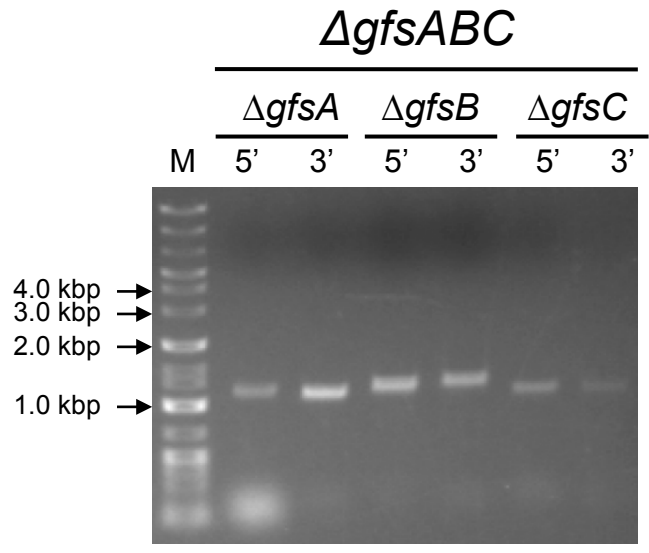

Fig. S5

Supplement: FIG S5 [file mSphere.00770-19-sf005.pdf]
