# Supplementary material for: Biosynthesis of β-(1→5)-Galactofuranosyl Chains of Fungal-Type and O-Mannose-Type Galactomannans within the Invasive Pathogen Aspergillus fumigatus
Source: mSphere. 2020 Jan 15;5(1):e00770-19. doi: 10.1128/mSphere.00770-19 (PMC6968653; doi:10.1128/mSphere.00770-19)
Supplement: TABLE S1 [file mSphere.00770-19-st001.pdf]

Table S1 *Aspergillus* strains used in this study

Strains	Genotype	Source of reference
A. fumigatus A1151	*KU80::pyrG*	da Silva Ferreira, 2006 (38); FGSC
A. fumigatus A1160	*KU80::pyrG, pyrG*$^{-}$	da Silva Ferreira, 2006 (38); FGSC
A. fumigatus ΔglfA	*KU80::AfpyrG, glfA::ptrA*	Komachi, 2013 (14)
A. fumigatus ΔgfsA	*KU80::AfpyrG, pyrG*$^{-}$*, AfgfsA::AnpyrG*	Komachi, 2013 (14)
A. fumigatus ΔgfsB	*KU80::AfpyrG, AfgfsB::ptrA*	This study
A. fumigatus ΔgfsC	*KU80::AfpyrG, pyrG*$^{-}$*, AfgfsC::AnpyrG*	This study
A. fumigatus ΔgfsAC	*KU80::AfpyrG, pyrG-, AfgfsA::AnpyrG, AfgfsC::ptrA*	This study
A. fumigatus ΔgfsABC	*KU80::AfpyrG, pyrG-, AfgfsA::AnpyrG, AfgfsB::hph, AfgfsC::ptrA*	This study
A. fumigatus ΔgfsA::A	*KU80::pyrG, AfgfsA::AnpyrG, ΔAfgfsA::AfgfsA-ptrA*	Katafuchi, 2017 (4)
A. fumigatus ΔgfsC::C	*KU80::AfpyrG, pyrG-, AfgfsC::AnpyrG, ΔAfgfsC::AfgfsC-ptrA*	This study
