# Supplementary material for: Biosynthesis of β-(1→5)-Galactofuranosyl Chains of Fungal-Type and O-Mannose-Type Galactomannans within the Invasive Pathogen Aspergillus fumigatus
Source: mSphere. 2020 Jan 15;5(1):e00770-19. doi: 10.1128/mSphere.00770-19 (PMC6968653; doi:10.1128/mSphere.00770-19)
Supplement: TABLE S2 [file mSphere.00770-19-st002.pdf]

Table S2 Oligonucleotides used in this study

Oligonucleotide primers	Sequence
AFUB_070620-1	5'- CCGTCGTCATTCACAGAGC -3'
AFUB_070620-2	5'- TCGTTACCAATGGGATCCCGTTAGATGTCGCCTGCTTGCAG -3'
AFUB_070620-3	5'- ATGCAAGAGCGGCTCATCGCGCCTTCTGTCCGAGTTCTTT -3'
AFUB_070620-2(*gfsB*::*hph*)	5'- GGAAGATCCAGGCACCGGGTTAGATGTCGCCTGCTTGCAG -3'
AFUB_070620-3(*gfsB*::*hph*)	5'- AAAACGCGTTTCGGGTTTACCCGCCTTCTGTCCGAGTTCTTT -3'
AFUB_070620-4	5'- TAGCCGGGGTGGAAATTCG -3'
AFUB_070620-7	5'- AGCTTTGAGCGTTTCTTGGG -3'
AFUB_070620-8	5'- GCTTCAGTGCCAACGAGAGTG -3'
AFUB_067290-1	5'- TTCGTAGTCGTTGGAACTCCTC -3'
AFUB_067290-2	5'- GGCGTGATAGCGTTGAAGGGGTATGTGTTCCGCTTTCCCTCG-3'
AFUB_067290-3	5'- AGGTTCCTTTGTGGCTGGGACCCCACACTGAGATGATTTGTGG-3'
AFUB_067290-2(*gfsC::ptrA*)	5'- TCGTTACCAATGGGATCCCGTATGTGTTCCGCTTTCCCTCG -3'
AFUB_067290-3(*gfsC::ptrA*)	5'- ATGCAAGAGCGGCTCATCGCCCCACACTGAGATGATTTGTGG -3'
AFUB_067290-4	5'- TGCCGCTTCTGTTCCTGTC -3'
AFUB_067290-7	5'- TTGTCTTTGCCACTGTCGTTTC -3'
AFUB_067290-8	5'- GCATATCCCATCCCCCATGAC -3'
AFUB_096220-1	5'- TACGCCGCTTGCTACTTGG -3'
AFUB_096220-4	5'- GCCAAATTCAATAGTGCACGC -3'
AfgfsC-complement-1	5'- GGAACTCCTCCAATTGTCTAATCG -3'
AfgfsC-complement-2	5'- TCGTTACCAATGGGATCCCCTACGCCATCTTCCAGATCAATTC -3'
AfgfsC-complement-3	5'- ATGCAAGAGCGGCTCATCGCCCTTCAACGCTATCACGCC -3'
AfgfsC-complement-4	5'- AGACATGATGGCGGTTCTCC -3'
AfgfsC-complement-7	5'- TTTGCCACTGTCGTTTCTCC -3'
AfgfsC-complement-8	5'- AAGCTGGAAGTGGGATGGCT -3'
pyrG-5	5'- CCCTTCAACGCTATCACGCC -3'
pyrG-6	5'- TCCCAGCCACAAAGGAACCT -3'
ptrA-5	5'- GGGATCCCATTGGTAACGA -3'
ptrA-6	5'- CGATGAGCCGCTCTTGCAT -3'
hph-5	5'- CCGGTGCCTGGATCTTCCT -3'
hph-6	5'- GGTAAACCCGAAACGCGTTTTAT -3'
pyrG-F	5'- GATCTACCCCTTGGAACGCA -3'
pyrG-R	5'- GACCATCGTGGGCAATTGGT -3'
ptrA-F	5'- CATATGTAAATGGCTGTGTCCCG -3'
ptrA-R	5'- TTTAGCTTTGACCGGTGAGC -3'
hph-F	5'- CCGCGGGATCCACTTAACG -3'
hph-R	5'- GTCTCTCCGCATGCCAGAAA -3'
pET50b-AfGfsB-F	5'- ATCATCATCATCATAGCTCCAGACCTGCTAGTCCCTC -3'
pET50b-AfGfsB-F	5'- GTACCGAGCTCCATATCACCCAGATGTAGGTTTCCAG -3'
pCold2-AfGfsC-F	5'- ATCATCATCATCATAGCCGCGCCGTGTATATGC -3'
pCold2-AfGfsC-R	5'- GTACCGAGCTCCATACTACGCCATCTTCCAGATC -3'
pET50b-Amp-F	5'- GAATTAATTCATGAGCGGATA -3'
pET50b-Amp-R	5'- AACACCCCTTGTATTACTGT -3'
Amp-gene-F	5'- AATACAAGGGGTGTTATGAGTATTCAACATTTCCGT -3'
Amp-gene-R	5'- CTCATGAATTAATTCTTACCAATGCTTAATCAGTG -3'
